# Supplementary material for: Homoharringtonine exhibits senotherapeutic activity that mitigates diet- and age-associated obesity and insulin resistance and extends lifespan in mice
Source: Nat Commun. 2026 Mar 31;17:2700. doi: 10.1038/s41467-026-70475-3 (PMC13039285; doi:10.1038/s41467-026-70475-3)
Supplement: Supplementary file 1 — Supplementary Information [file 41467_2026_70475_MOESM1_ESM.pdf]

Homoharringtonine exhibits senotherapeutic activity that mitigates diet- and age-associated obesity and insulin resistance and extends lifespan in mice

Eok-Cheon Kim[1,2#], Han-Byul Jung[3#], Yu-kyoung Park[1,3], Youlim Son[1,2], Hye-Na Cha[1,3], Yash Patel[4], Ju Hee Lee[4,5], Minah Choi[2], Soyoung Park[1,3], Il-Kug Kim[1,6], Lauren Pickel[4], Seungju Lee[7], Yuna Ha[7], Min-Gyeong Shin[3], Qiwei Zhang[4,5], Jielin Yang[4], Bruno Rodrigues de Oliveira[4], Nathaniel Vo[4,5], Annie Yew[4,5], Jacques Togo[4], Kafi N. Ealey[4], Su-Ryun Jung[1,3], Sunjin Moon[1], Hye-Jin Yoon[1,2], Jee-Young Lee[7], Hoon-Ki Sung[4,5*], Jae-Ryong Kim[1,2*], So-Young Park[1,3*]

Supplementary information

Pharmacodynamic comparison of HHT with ABT263

To compare ABT263 with HHT, non-senescent and senescent cells were treated with ABT263 or HHT with increasing concentrations and determined the values of median lethal dose (LD50) in non-senescent cells, median effective dose (ED50) in senescent cells, and therapeutic index (TI) (Fig. S4 and Supplementary Table 3). Our results indicate that ABT263 exhibits senolytic activity across all senescent cell types tested. In contrast, HHT demonstrates senolytic activity in HPAs and HDFs, but exhibits senomorphic activity in HUVECs and hRPEs, suggesting a cell type-specific distinction between senolytic and senomorphic activities of HHT (Fig. S4).

Supplementary Figures

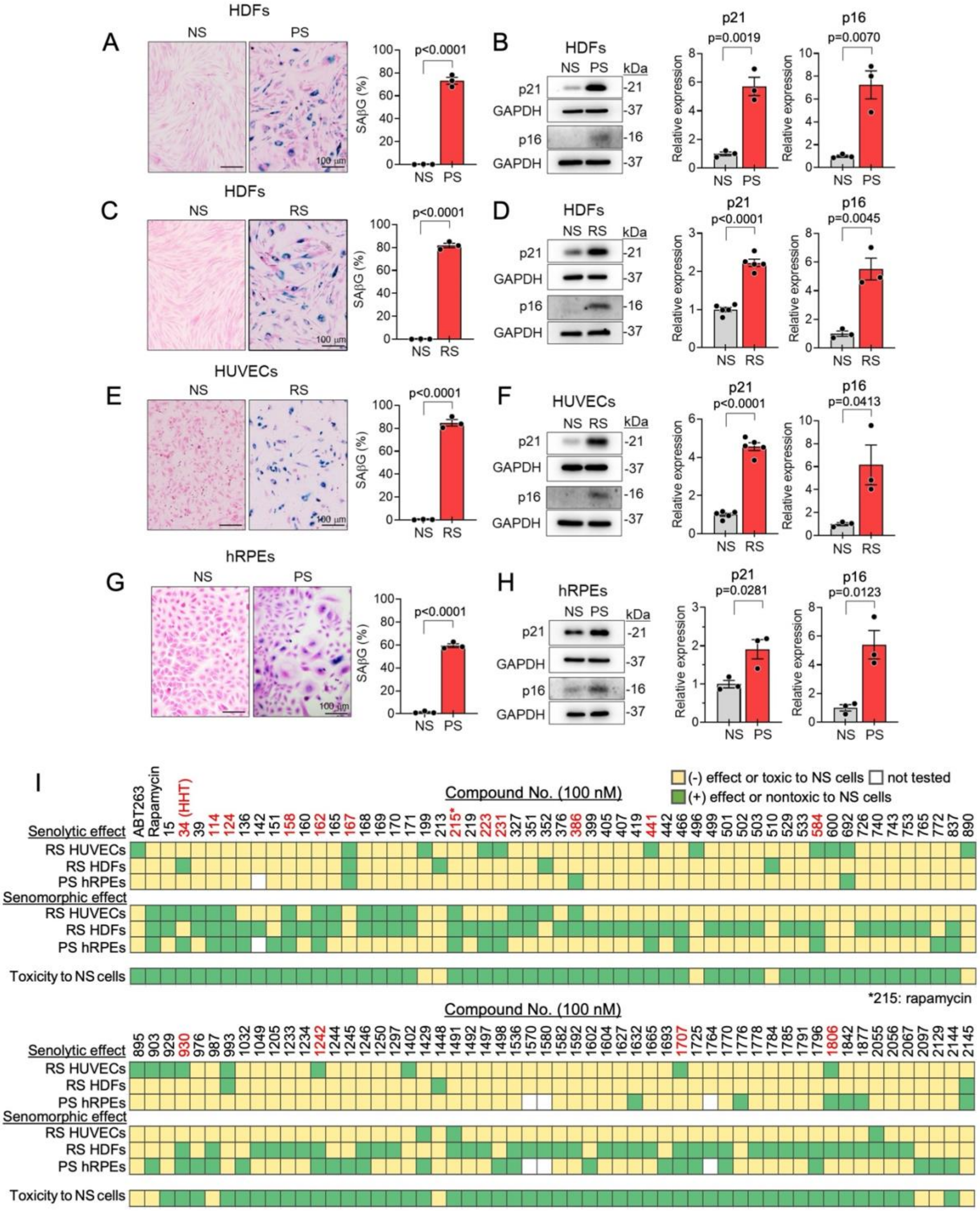

Supplementary Figure 1. Validation of senescence in cells used in the primary and secondary screening, and the secondary screening results with 110 compounds. Senescence was induced in various cell types by either doxorubicin (Dox) treatment, defined as prematurely senescent (PS) cells, or by serial passaging, defined as replicatively senescent

(RS) cells. All cells were cultured in medium containing 10% FBS. Cellular senescence was evaluated by SAβG staining and by assessing the protein expression levels of p21 and p16 (A-H). **A** and **B.** Non-senescent (NS) and PS HDFs. **C** and **D.** NS and RS HDFs. **E** and **F.** NS and RS HUVECs. **G** and **H.** NS and PS hRPEs. After the primary screening of 2,150 compounds, we selected 110 and conducted secondary screening in RS HDFs, RS HUVECs, and PS hRPEs. ABT263 and rapamycin were used as positive controls for senolytic, and senomorphic, respectively. **I**. Senolytic and senomorphic effects of 110 compounds in RS HUVECs, RS HDFs, and PS hRPEs, and their cytotoxicity in NS cells. Compounds in red were selected senotherapeutic candidates. Representative images were shown, and values are presented as means ± SEM of three independent experiments. Data were analyzed with two-tailed Student's *t* test in three independent experiments. HDFs, human dermal fibroblasts; hRPEs, human retina pigment epithelial cells; HUVECs, human umbilical vein endothelial cells; SAβG, senescence-associated β-galactosidase.

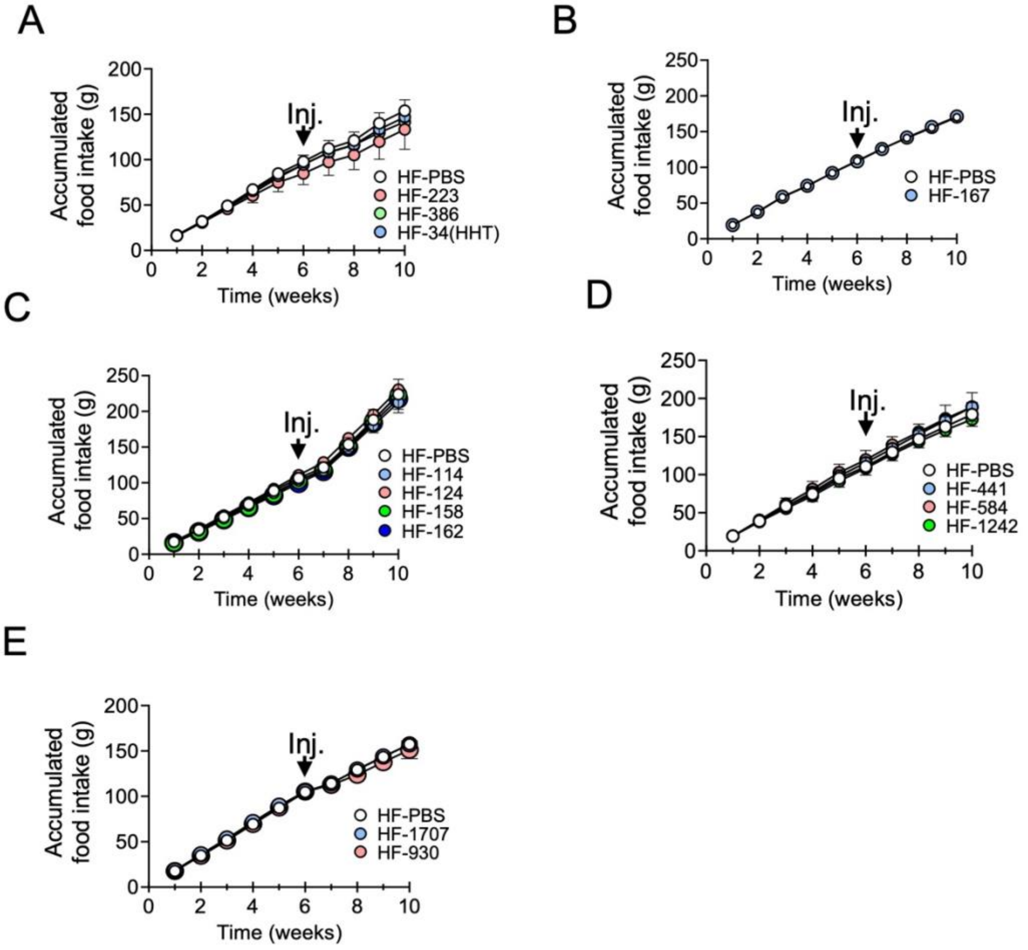

Supplementary Figure 2. **Effects of 13 senotherapeutic candidates on the food intake of high-fat diet (HF)-induced obese mice**. After feeding HF (60% fat) for 6 weeks, mice were administered with candidate compounds for 6 weeks while maintaining the mice on HF. Food intake was measured manually once a week. **A**. Mice treated with phosphate-buffered saline (PBS), compound 223, compound 386, and 34 (homoharringtonine, HHT). **B**. Mice treated with PBS and compound 167. **C**. Mice treated with PBS, compound 114, compound 124, compound 158, and compound 162. **D**. Mice treated with PBS, compound 441, compound 584, and compound 1242. **E**. Mice treated with PBS, compound 1707, and compound 930. Inj., start intraperitoneal injection of senotherapeutic candidates three times per week. Values are presented as means ± SEM.

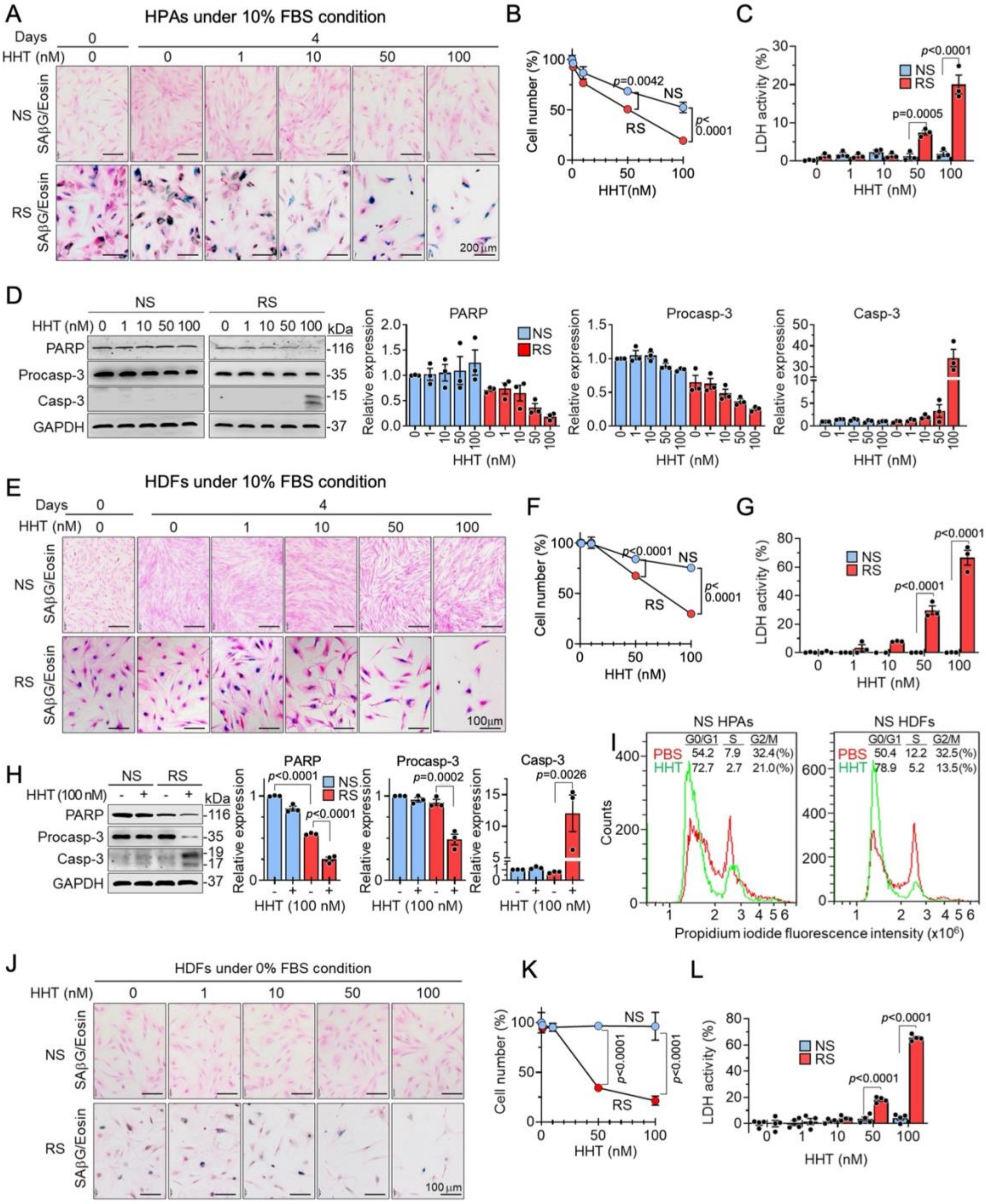
A
HPAs under 10% FBS condition
Days
0
4
HHT (nM)
0
0
1
10
50
100
NS
SAβG/Eosin
RS
SAβG/Eosin
200 μm
B
Cell number (%)
p=0.0042
NS
RS
p<
0.0001
HHT(nM)
C
LDH activity (%)
p<0.0001
NS
RS
p=0.0005
HHT (nM)
D
NS
RS
HHT (nM) 0 1 10 50 100 0 1 10 50 100 kDa
PARP
-116
Procasp-3
-35
Casp-3
-15
GAPDH
-37
PARP
Procasp-3
Casp-3
Relative expression
HHT (nM)
E
HDFs under 10% FBS condition
Days
0
4
HHT (nM)
0
0
1
10
50
100
NS
SAβG/Eosin
RS
SAβG/Eosin
100μm
F
Cell number (%)
p<0.0001
NS
p<
0.0001
RS
HHT (nM)
G
LDH activity (%)
p<0.0001
NS
RS
p<0.0001
HHT (nM)
H
NS
RS
HHT (100 nM)
kDa
PARP
-116
Procasp-3
-35
Casp-3
-19
-17
GAPDH
-37
PARP
p<0.0001
NS
RS
p<0.0001
Procasp-3
p=0.0002
Casp-3
p=0.0026
Relative expression
HHT (100 nM)
I
NS HPAs
G0/G1 S G2/M
PBS 54.2 7.9 32.4(%)
HHT 72.7 2.7 21.0(%)
NS HDFs
G0/G1 S G2/M
PBS 50.4 12.2 32.5(%)
HHT 78.9 5.2 13.5(%)
Counts
Propidium iodide fluorescence intensity (x10^6)
J
HDFs under 0% FBS condition
HHT (nM)
0
1
10
50
100
NS
SAβG/Eosin
RS
SAβG/Eosin
100 μm
K
Cell number (%)
NS
p<0.0001
p<0.0001
RS
HHT (nM)
L
LDH activity (%)
p<0.0001
NS
RS
p<0.0001
HHT (nM)

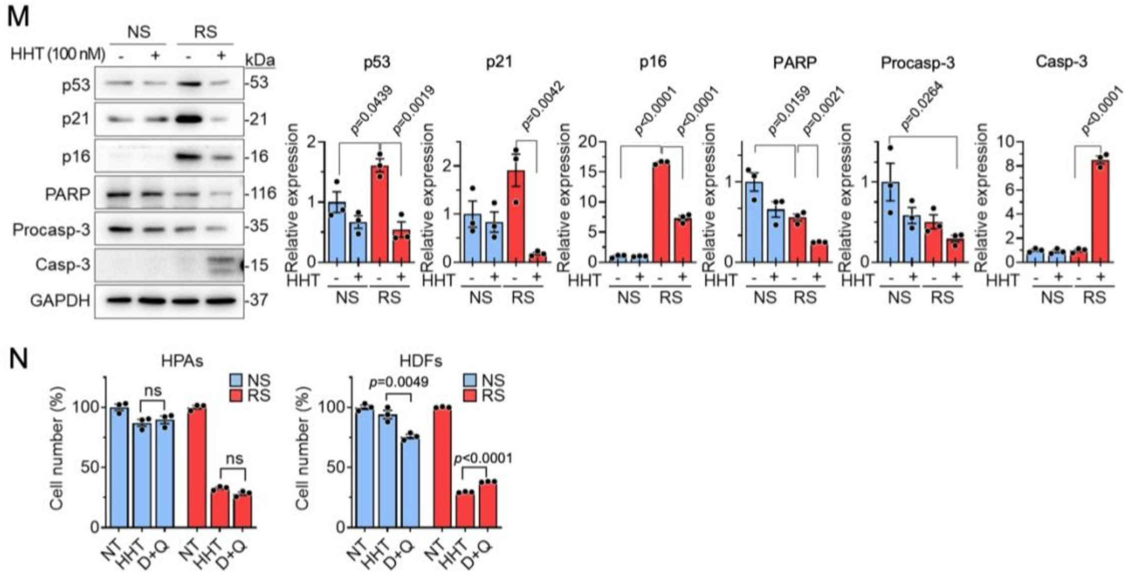

Supplementary Figure 3. Senotherapeutic effect of HHT in replicatively senescent HDFs and HPAs. Non-senescent (NS) and replicatively senescent (RS) human visceral preadipocytes (HPAs) were cultured in medium containing 10% FBS and treated with the indicated concentrations of HHT for 4 days (A-D). **A**. Representative images of SAβG and eosin staining of HPAs. **B**. Cell number of HPAs measured by cell counting at the indicated HHT concentrations (n=6 per group). **C**. Lactate dehydrogenase (LDH) activity in the culture medium of HPAs (n=3 per group). **D**. Expression levels and quantification of apoptosis-associated proteins in HPAs (n=3 per group). NS and RS human dermal fibroblasts (HDFs) were cultured in medium containing 10% FBS and treated with indicated concentrations of HHT for 4 days (E-H). **E**. Representative images of SAβG and eosin staining of HDFs. **F**. Cell number of HDFs measured by cell counting at the indicated HHT concentrations (n=3 per group). **G**. Lactate dehydrogenase (LDH) activity in the culture medium of HDFs (n=3 per group). **H**. Expression levels and quantification of apoptosis-associated proteins in HDFs (n=3 per group). **I**. Cell cycle analysis by flow cytometry in NS HPAs and NS HDFs cultured with 10% FBS and treated with or without 100 nM HHT for 2 days (n=1 per group). NS and RS HDFs were cultured in medium containing 0% FBS and treated with indicated concentrations of HHT for 4 days (**J-M**). **J**. Representative images of SAβG and eosin staining of HDFs. **K**. Cell number of HDFs measured using a CCK-8 assay at the indicated HHT concentrations (n=3 per group). **L**. Lactate dehydrogenase (LDH) activity in the culture medium of HDFs (n=4 per group). **M**. Expression levels and quantification of senescence- and apoptosis-associated proteins in HDFs (n=3 per group). **N**. Cell number of HPAs and HDFs treated with 100 nM HHT or 200 nM dasatinib + 30 μM quercetin (D+Q) in 10% FBS containing medium for 4

days (n=3 per group). Values are means ± SEM. Data were analyzed via one-way ANOVA followed by a post-hoc test. CCK-8, Cell Counting Kit-8; HHT, homoharringtonine; NT, not treated; SAβG, senescence-associated β-galactosidase.

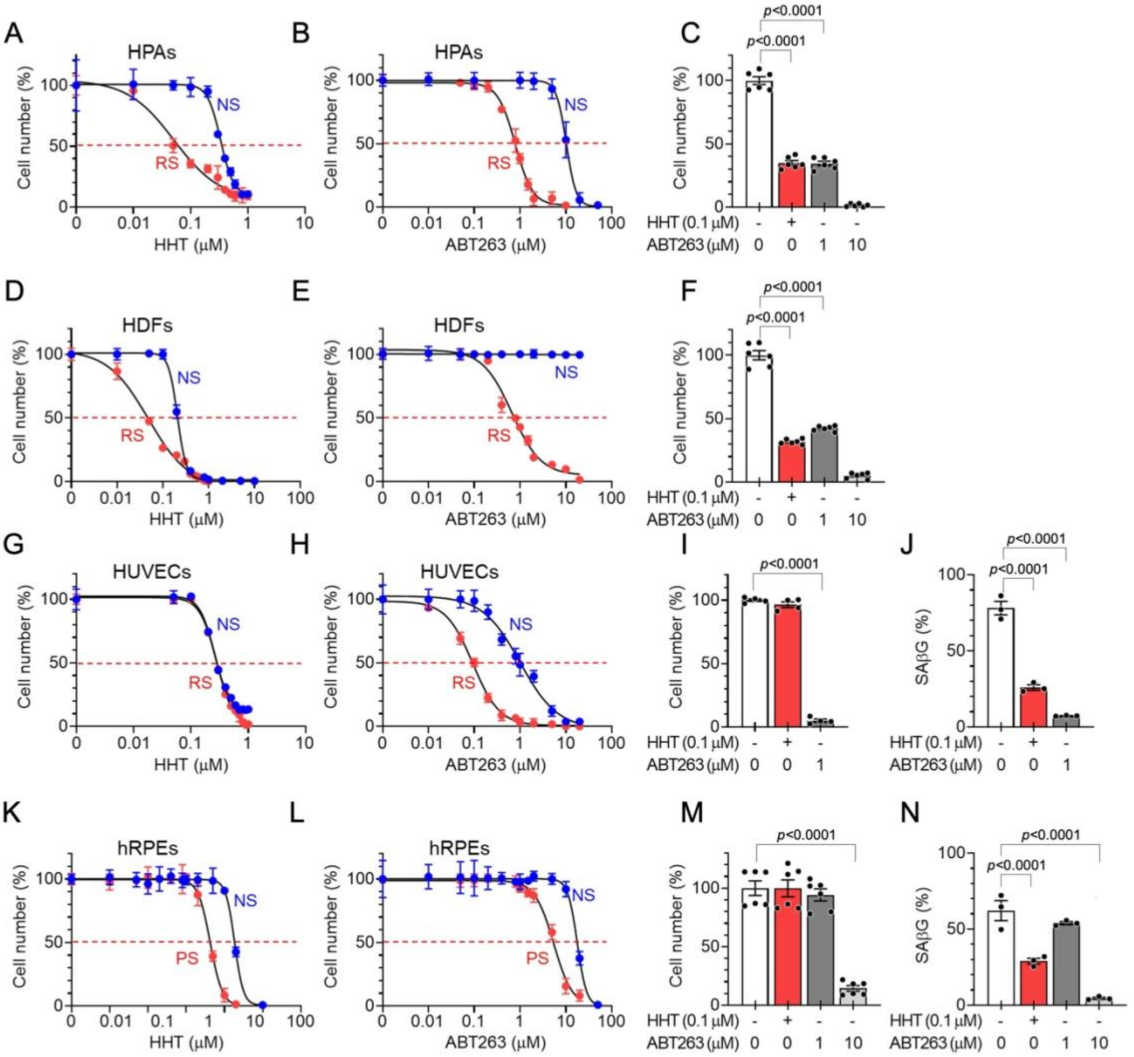

Supplementary Figure 4. **Comparison of senotherapeutic effects of HHT and ABT263**. After non-senescent (NS) and senescent cells were treated with increasing concentrations of HHT and ABT263, dose-response curves for cell survival were obtained by measuring cell number using a CCK-8 assay. Senescence was induced by treatment with doxorubicin in hRPEs, defined as prematurely senescent (PS) cells, and by serial passaging in other cells, defined as replicatively senescent (RS) cells. Cell number and the percentages of SAβG positive cells in senescent cells were compared at 100 nM HHT, and 1 and 10 μM of ABT263. **A.** Dose-response curves of HHT in human visceral preadipocytes (HPAs). **B.** Dose-response curves of ABT263 in HPAs. **C.** Cell number in RS HPAs (n=6 in each group). **D.** Dose-response curves of HHT in human dermal fibroblasts (HDFs). **E.** Dose-response curves of ABT263 in HDFs. **F.** Cell number in RS HDFs (n=6 in each group). **G.** Dose-response curves of HHT in human umbilical vein endothelial cells (HUVECs). **H**. Dose-response curves of ABT263 in HUVECs. **I**. Cell number in RS HUVECs (n=4 in each group). **J**. The percentages

of SAβG-positive cells in RS HUVECs (n=3 in each group). **K**. Dose-response curves of HHT in human retina pigment epithelial cells (hRPEs). **L**. Dose-response curves of ABT263 in hRPEs. **M**. Cell number in PS hRPEs (n=6 in each group). **N**. The percentages of SAβG positive cells in PS hRPEs (n=3 in each group). Values are means ± SEM. Data were analyzed via one-way ANOVA followed by a post-hoc test. CCK-8, Cell Counting Kit-8; HHT, homoharringtonine; SAβG, senescence-associated β-galactosidase.

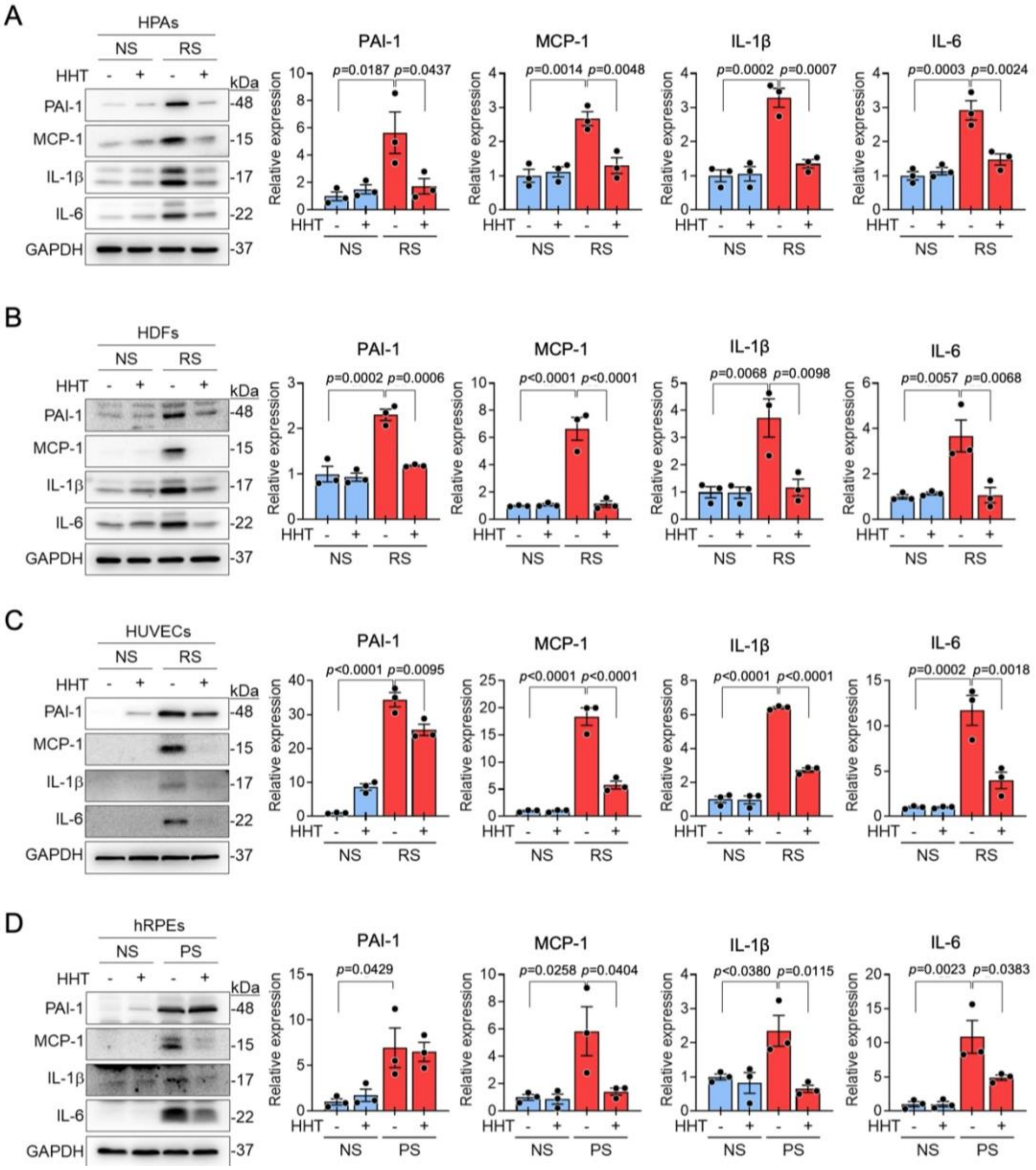

Supplementary Figure 5. **Effects of HHT on the levels of SASP proteins in non-senescent and senescent cells.** Senescence was induced by treatment with doxorubicin in hRPEs, defined as prematurely senescent (PS) cells, and by serial passaging in other cells, defined as replicatively senescent (RS) cells. Cells were cultured in medium containing 10% FBS. After treatment of non-senescent (NS) and senescent cells with 100 nM HHT for 4 days, cells and culture media were harvested. The expression levels of PAI-1, MCP-1, IL-1β, and IL-6 proteins in media and GAPDH in cell lysates were measured by Western blotting. **A**. Expression levels and quantification of SASP proteins in HPAs. **B**. Expression levels and quantification of SASP proteins in HDFs. **C**. Expression levels and quantification of SASP proteins in HUVECs. **D**. Expression levels and quantification of SASP proteins in hRPEs. Representative images were

shown and values are means ± SEM of three independent experiments. Data were analyzed via one-way ANOVA followed by a post-hoc test. HHT, homoharringtonine; IL-1β, interleukin-1β; IL-6, interleukin 6; MCP1, monocyte chemoattractant protein-1; HDFs, human dermal fibroblasts; HPAs, human visceral preadipocytes; hRPEs, human retina pigment epithelial cells; HUVECs, human umbilical vein endothelial cells; PAI-1, plasminogen activator inhibitor-1, SAβG, senescence-associated β-galactosidase; SASP, senescence-associated secretory phenotypes.

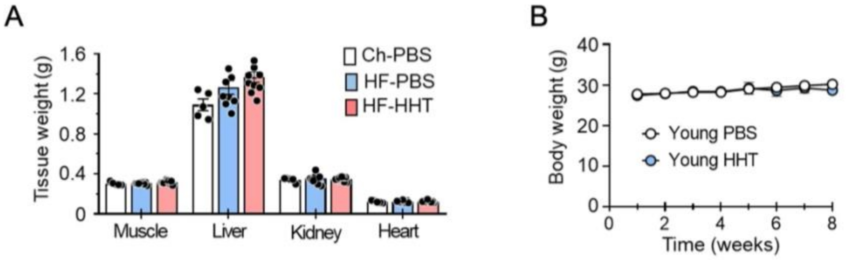

Supplementary Figure 6. Effects of HHT on body weight and tissue weight. After feeding high-fat diet (HF) for 6 weeks, mice were administered with HHT (HF-HHT) or PBS (HF-PBS) for 8 weeks while they were kept on HF. Chow diet-fed mice were kept on a chow diet during the experimental period and injected with PBS for 8 weeks (Ch-PBS). **A**. Effects of HHT on the tissue weights of HF-induced obese mice (n=5 for Ch-PBS; n=9 for HF-PBS; n=10 for HF-HHT). **B**. Change of body weight during HHT administration in chow diet-fed young mice (n=5 for Young PBS; n=4 for Young HHT). Values are presented as means ± SEM. Data were analyzed via one-way ANOVA followed by a post-hoc test in **A** and with two-tailed Student's *t* test in **B**. HHT, homoharringtonine; PBS, phosphate-buffered saline.

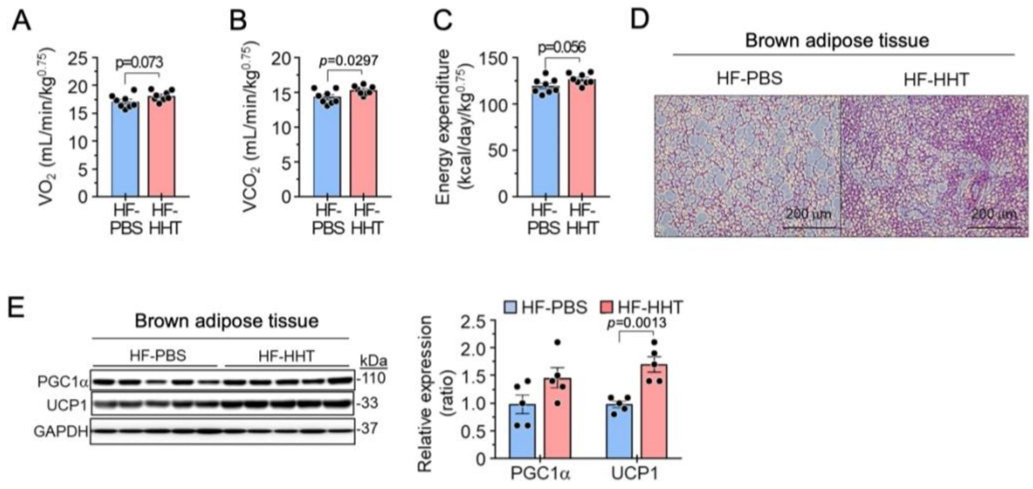

Supplementary Figure 7. **Effects of HHT on the thermogenesis of brown adipose tissues in HF-fed obese mice**. After feeding high-fat diet (HF) for 6 weeks, mice were administered with HHT or PBS for 8 weeks while the mice were kept on HF. Metabolic cage analysis (A-C) (n=8 in each group). During the measurement period, all mice were provided *ad libitum* access to food and water to maintain normal physiological conditions. **A**. VO_2. **B**. VCO_2. **C**. Energy expenditure. **D**. Histological analysis of brown adipose tissues after staining with hematoxylin and eosin. Scale bar, 200 μm. **E**. Expression levels of PGC1α and UCP1 proteins in the brown adipose tissues (n=5 in each group). Values are presented as means ± SEM. Data were analyzed with two-tailed Student's *t* test. GAPDH, glyceraldehyde 3-phosphate dehydrogenase; HHT, homoharringtonine; PBS, phosphate buffered saline; PGC1α, Peroxisome proliferator-activated receptor γ coactivator 1-α; UCP1, uncoupling protein 1.

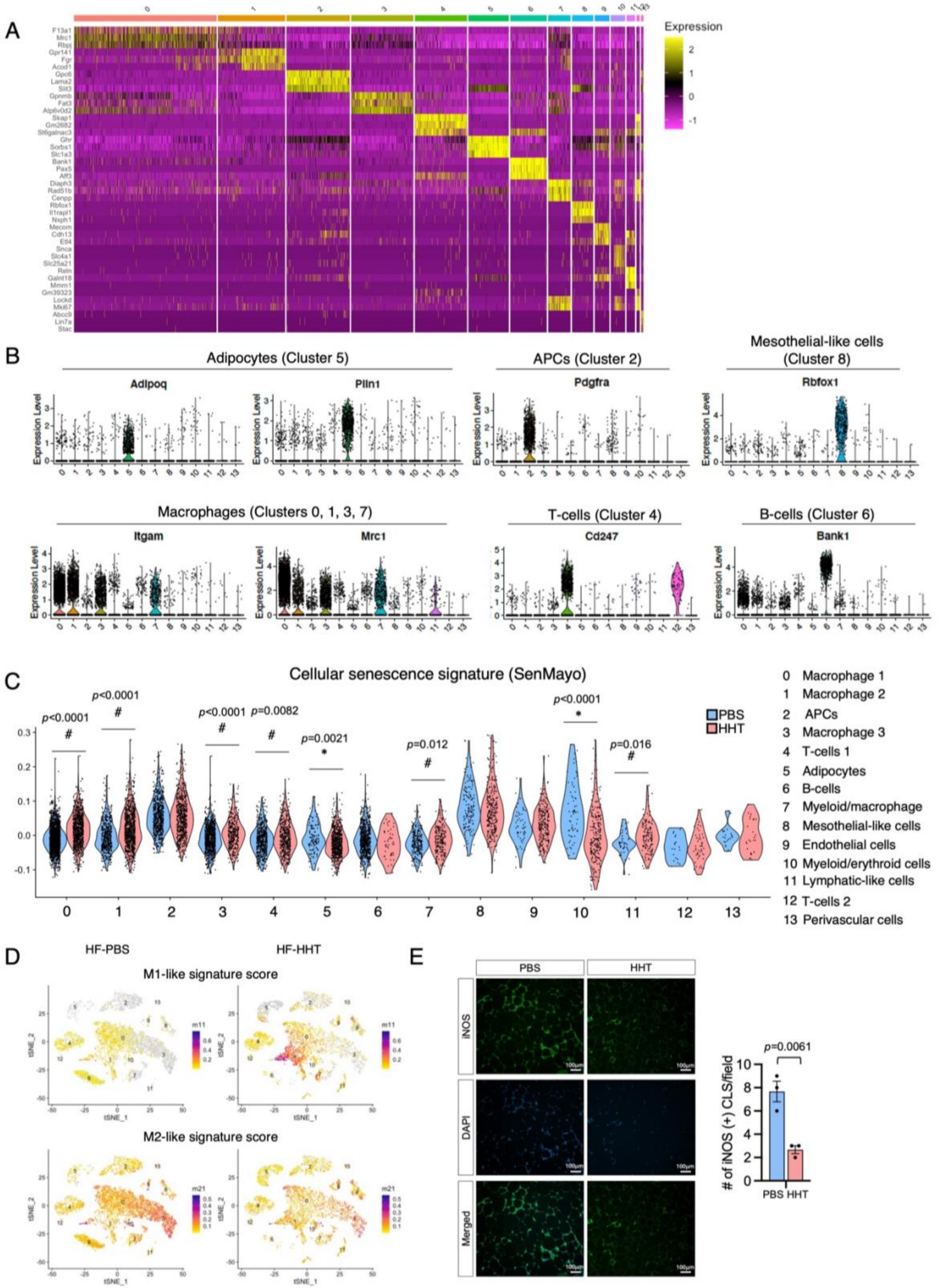

Supplementary Figure 8. Normalized expression values of the representative genes of cell clusters. After feeding high-fat diet (HF) for 6 weeks, male mice were administered with HHT

or PBS for 8 weeks while they were kept on HF. Pooled epididymal fat was analyzed with single nucleus RNA sequencing (snRNA-seq). **A.** Heatmap of top 3 specific genes enriched in each cell population. **B**. Representative gene expression of major cell clusters including mature adipocyte (Cluster 5), adipocyte precursor cells (APCs), mesothelial-like cells, macrophages, T- and B-cells. **C.** SenMayo signature in various cell clusters. **D**. M1- and M2-like gene signature score. **E**. Staining and quantification of iNOS positive crown-like structure (n=3 in each group). Data were analyzed with two-tailed Student's *t* test. *, a statistically significant decrease in HHT compared to PBS; #, a statistically significant increase in HHT compared to PBS. APCs, adipose precursor cells; CLS, crown-like structure; DAPI, 4',6-diamidino-2-phenylindole; HF, high fat diet; HHT, homoharringtonine; iNOS, inducible nitric oxide synthase; PBS, phosphate buffered saline.

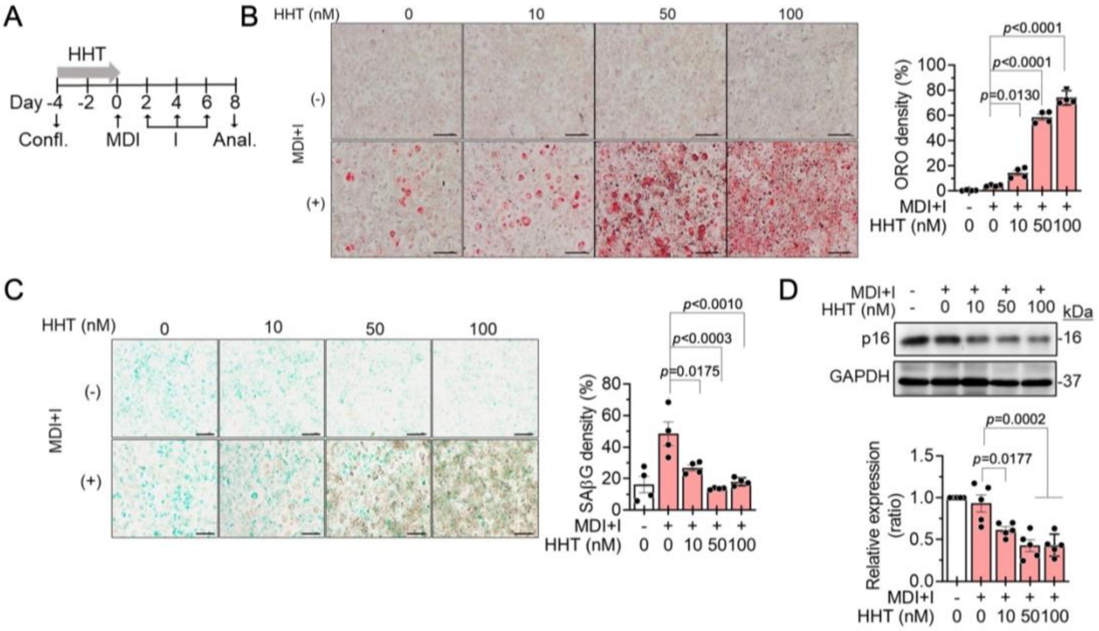

Supplementary Figure 9. Senotherapeutic effect of HHT on the differentiation of senescent 3T3-L1 preadipocytes. After treatment of confluent 3T3-L1 cells (> passage 35) with the indicated concentration of HHT for 4 days, cells were treated with or without MDI+I (A-D). **A**. Experimental scheme. **B**. Representative image of oil-red O staining (ORO) and its quantification (n=4 in each group). **C**. Representative image and quantification of SAβG staining (n=4 in each group). **D**. Expression levels of p16 protein (n=5 in each group). Values are presented as means ± SEM. Data were analyzed via one-way ANOVA followed by a post-hoc test. HHT, homoharringtonine; I, Insulin; MDI, Isobutylmethyl xanthine (IBMX), dexamethasone and insulin; LDH, lactate dehydrogenase; SAβG, senescence-associated β-galactosidase.

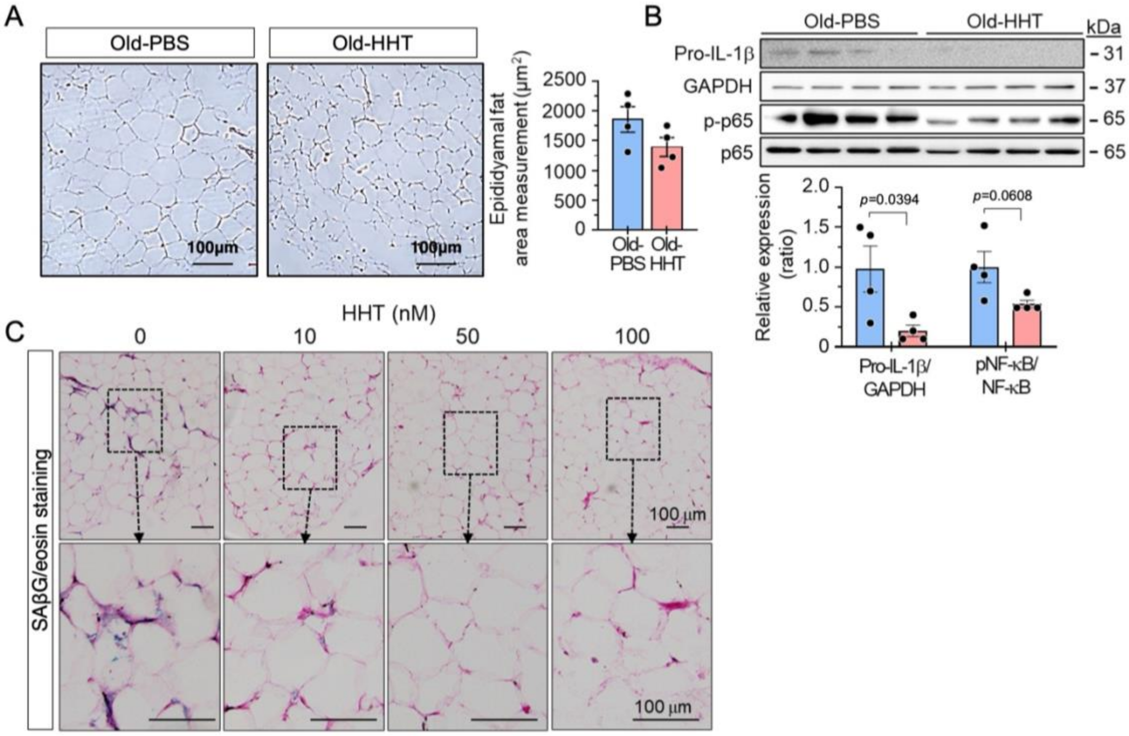

Supplementary Figure 10. Effect of HHT on adipose tissue senescence in aged mice and humans. Eighteen-month-old mice were administered with HHT or PBS three times a week for 13 weeks (A-B) (n=4 in each group). **A.** Histological analysis of epididymal fat after staining with hematoxylin and eosin and measurement of adipocyte size. Scale bar, 100 μm **B.** Expression levels and quantification of pro-IL-1β, phosphorylated p65 (p-p65), and p65 proteins in the epididymal fat. Values are presented as means ± SEM. Data were analyzed with two-tailed Student's *t* test. **C**. SAβG and eosin staining in the human subcutaneous adipose tissue sections after *ex vivo* culture and treatment with the indicated concentrations of HHT. Scale bar, 100 μm. GAPDH, glyceraldehyde 3-phosphate dehydrogenase; HHT, homoharringtonine; IL-1β, interleukin-1β; PBS, phosphate buffered saline; SAβG, senescence-associated β-galactosidase.

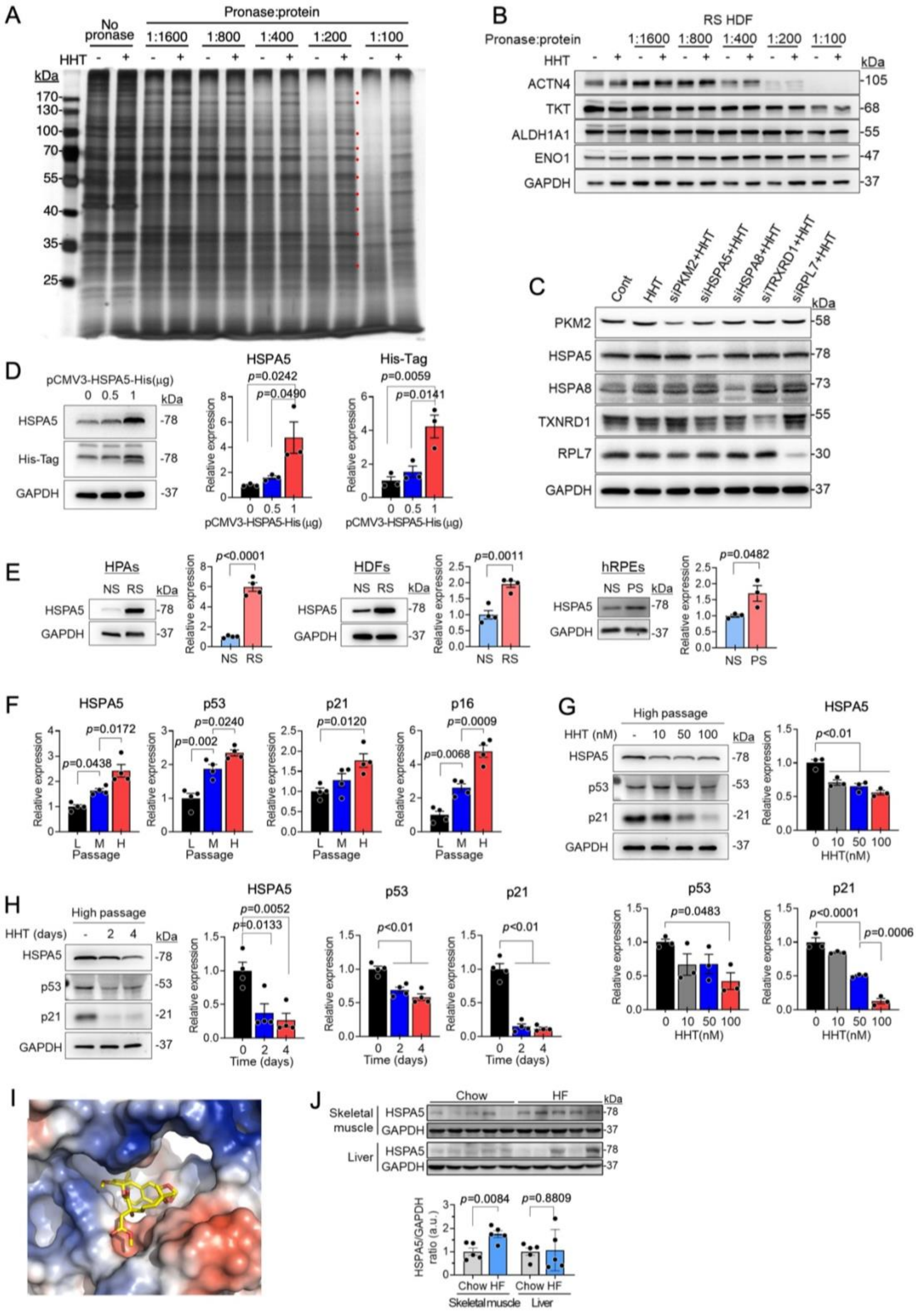

Supplementary Figure 11. Identification of HSPA5 as a potential target of senotherapeutic activity of HHT. **A**. Representative image of silver staining gel of RS HDFs in drug affinity responsive target stability (DARTS) assay**. B**. Western blotting of five other

proteins identified following DARTS assay in RS HDFs. **C**. Expression levels of five proteins in HPAs transfected with siRNAs, incubated for 1 day, and then treated with 100 nM HHT for 2 days, analyzed by western blotting. **D**. Expression levels and quantification of HSPA5 and His-tag in HPAs transfected with pCMV3-HSPA5-His plasmids (n=3 per group). **E**. Expression levels and quantification of HSPA5 protein in HPAs, HDFs, and hRPEs (n=4 in HPAs and HDFs, n=3 in hRPEs). HPAs in 10% FBS containing medium were cultured by serial passaging and harvested at passages 2-3 (low, L), 6-7 (mid, M), and 12-13 (high, H). Following treatment with increasing concentrations of HHT for indicated times, the expression levels of several proteins were measured by Western blotting (**F-H**). **F**. Quantification of HSPA5, p53, p21, and p16 proteins in three different states of HPAs (n=4 per group). **G**. Expression levels and quantification of HSPA5, p53, and p21 proteins in high passage HPAs treated with the indicated concentrations of HHT for 4 days (n=3 per group). **H**. Expression levels and quantification of HSPA5, p53, and p21 proteins in high passage HPAs treated with 100 nM HHT for the indicated times (n=4 per group). **I**. Surface representation of HSPA5 with HHT. Surface colors show electrostatic charge, with red indicating negatively charged regions and blue indicating positively charged regions. **J**. Western blot analysis and quantification of HSPA5 expression in skeletal muscle (tibialis anterior), and liver from chow-fed and HF-induced obese mice (n=5 per group). Values are means ± SEM. Data were analyzed via one-way ANOVA followed by a post-hoc test or two-tailed Student's *t* test. FBS, fetal bovine serum; HDFs, human dermal fibroblasts; HF, high fat diet; HPAs; human visceral preadipocytes; hRPEs, human retinal pigment epithelial cells; NS, non-senescent; RS, replicatively senescent.

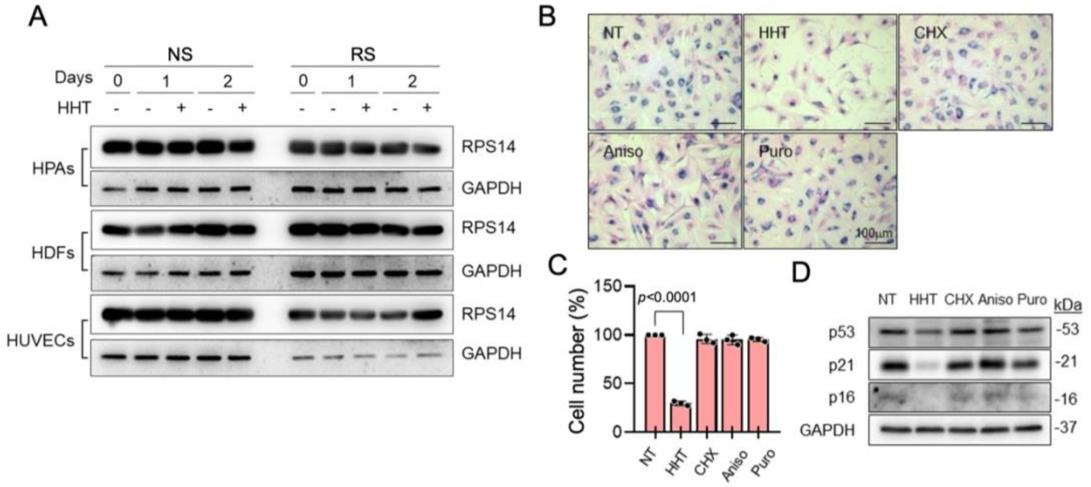

Supplementary Figure 12. Effect of HHT on the expression of ribosomal protein and comparison of senotherapeutic effect of HHT with translation inhibitors. **A.** Non-senescent (NS) and senescent cells were treated with 100 nM HHT for the indicated times and the expression level of RPS14 protein was measured by Western blotting. Replicatively senescent human visceral preadipocytes (RS HPAs) were treated with 100 nM HHT, 100 nM cycloheximide (CHX), 100 nM anisomycin (Aniso), and 100 nM puromycin (Puro) for 4 days, and then cell number, SAβG staining, and the expression levels of p53, p21, and p16 proteins were measured (B-D). **B**. Representative images of cells stained with SAβG and eosin. **C**. Cell number measured by cell counting (n=3 in each group). **D**. Expression levels of p53, p21, and p16 proteins. Values are means ± SEM. Data were analyzed via one-way ANOVA followed by a post-hoc test. HHT, homoharringtonine; HDFs, human dermal fibroblasts; HUVECs, human umbilical vein endothelial cells; NT, not treated; RPS14, ribosomal protein S14; SAβG, senescence-associated β-galactosidase.

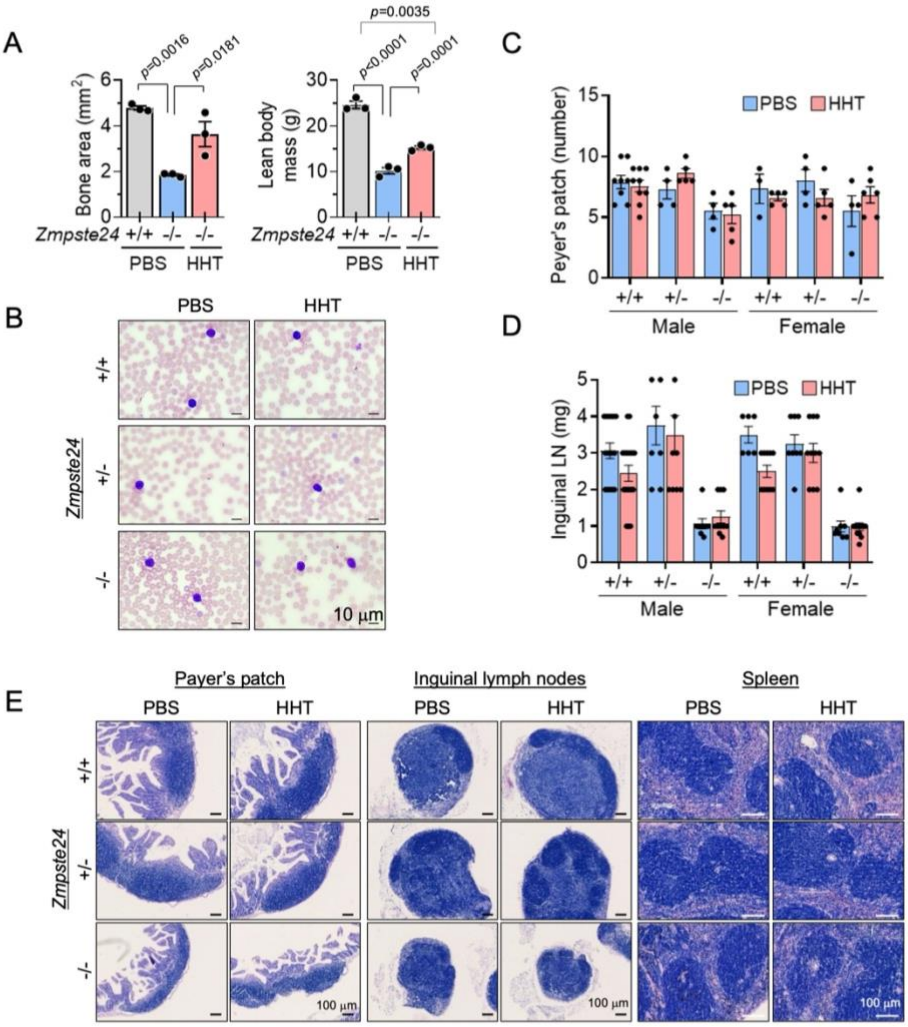

Supplementary Figure 13. Effect of HHT on tissue composition and histology of lymphatic tissue in *Zmpste24*$^{-/-}$ mice. *Zmpste24*$^{+/+}$, *Zmpste24*$^{+/-}$, and *Zmpste24*$^{-/-}$ male mice at 5 weeks old were intraperitoneally injected with vehicle (PBS) and HHT for 10 weeks, and then blood and tissues were harvested. **A.** Bone area and lean body mass analyzed by DEXA (n=3 in each group). **B**. Giemsa stain of blood smear. **C**. Numbers of Peyer's patches. **D**. Weight of inguinal lymph nodes. **E**. Histological analysis of Peyer's patch, inguinal lymph nodes, and spleen. Scale bar, 100 μm. Values are presented as means ± SEM. Data were analyzed via one-way analysis of variance (ANOVA) followed by a post-hoc test or via two-tailed Student's *t* test. DEXA, dual-energy X-ray absorptiometry; HHT, homoharringtonine; SAβG, senescence-associated β-galactosidase.

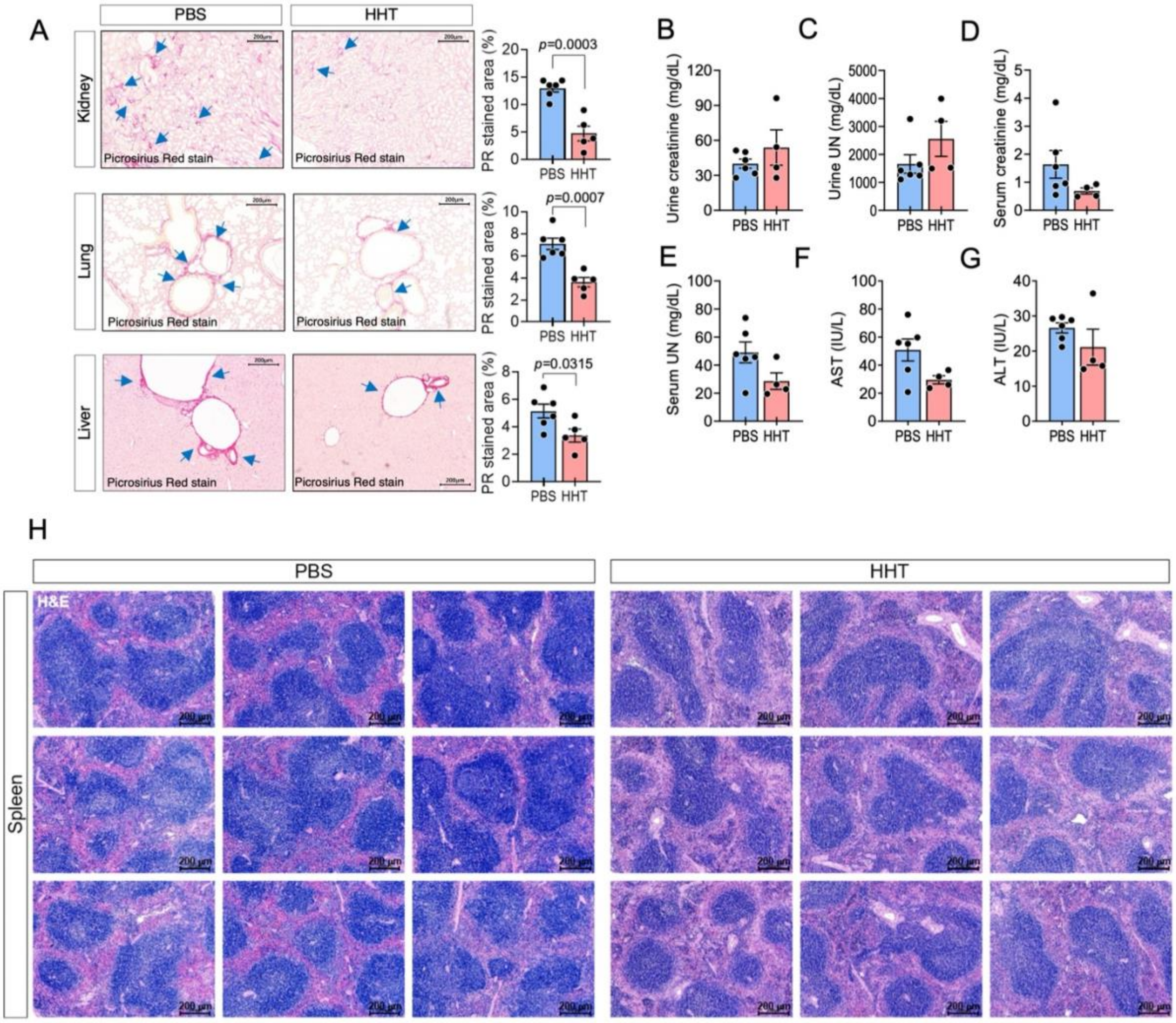

Supplementary Figure 14. Effects of HHT on tissue fibrosis, renal and hepatic toxicity, and spleen histology in physiologically aged mice. Male C57BL/6N mice at 16 months (n=8 in each group) were intraperitoneally injected with PBS or HHT once a week for 12 months. **A**. Picrosirius Red (PR) staining and its quantification of tissue sections from the lung, kidney, and liver. (n=6 in PBS and n=5 in HHT). **B**. Urine creatinine (n=6 in PBS and n=4 in HHT). **C**. Urine urea nitrogen (UN) (n=6 in PBS and n=4 in HHT). **D**. Serum creatinine (n=6 in PBS and n=4 in HHT). **E**. Serum UN (n=6 in PBS and n=4 in HHT). **F**. Serum aspartate aminotransferase (AST, n=6 in PBS and n=4 in HHT). **G**. Serum alanine aminotransferase (ALT, n=6 in PBS and n=4 in HHT). **H.** Hematoxylin and eosin staining of tissue sections from the spleen. Values are presented as means ± SEM. Data were analyzed with two-tailed Student's *t* test. HHT, homoharringtonine.

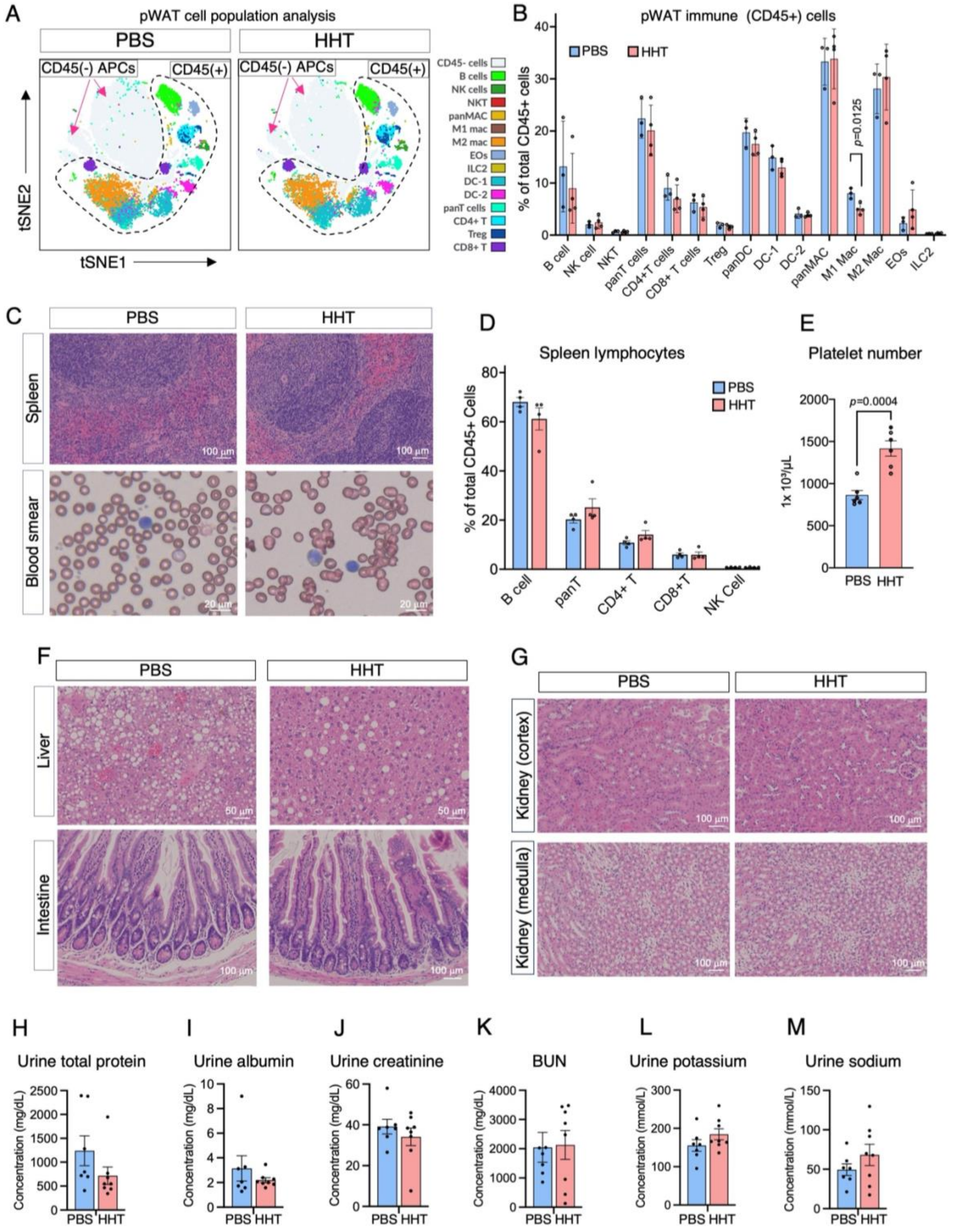

Supplementary Figure 15. Effects of HHT on adipose tissue cellular changes and tissue toxicity in high-fat diet (HF)-fed aged mice. Two-month-old male C57BL/6J mice were fed HF for 11 months and injected with HHT once a week for 5 months. **A.** Stromal cell analysis of perigonadal white adipose tissue (pWAT) using mass cytometry (CyTOF). **B.** CyTOF analysis of pWAT immune cell populations. **C.** Hematoxylin and eosin staining of spleen tissue

(top panel) and Giemsa stain of blood smear (bottom panel). **D**. CyTOF analysis of spleen immune cells (n=4 per group). **E.** Platelet count (n=6 per group). **F.** Hematoxylin and eosin staining of the liver (top panel) and small intestine (bottom panel) tissue. **G.** Hematoxylin and eosin staining of kidney cortex (top panel) and medulla (bottom panel). **H.** Urine total protein concentration. **I.** Urine albumin concentration. **J.** Urine creatinine concentration. **K.** Blood urea nitrogen (BUN). Urine potassium (**L**) and sodium (**M**) concentration (n=7-8 per group). Values are presented as means ± SEM. Data were analyzed with two-tailed Student's *t* test. HHT, homoharringtonine; PBS, phosphate buffered saline.

Supplementary Tables

Supplementary Table 1. Evaluation of senolytic or senomorphic activity of 15 compounds in three senescent cell types cultured in 10% FBS containing medium

Compound No.	Conc. (nM)	Senolytic activity			Senomorphic activity		
		RS HUVECs	RS HDFs	PS hRPEs	RS HUVECs	RS HDFs	PS hRPEs
34 (HHT)	100	-	+	-	+	-	+
223	100	+	-	-	-	+	+
114	100	-	-	-	+	+	+
124	100	-	-	-	+	+	+
158	100	-	-	-	+	+	+
162	100	-	-	-	+	+	+
167	100	+	+	+	-	-	-
231	100	+	-	-	-	+	+
386	100	-	-	+	+	+	-
441	100	+	-	-	-	+	+
584	100	+	-	-	-	+	+
930	100	+	-	-	-	+	+
1242	100	+	-	-	-	+	+
1707	100	+	-	-	-	+	+
1806	100	+	-	+	-	+	-

+, present; -, absent. Conc, concentration; HDFs, human dermal fibroblasts; HHT, homoharringtonine; hRPEs, human retinal pigment epithelial cells; HUVECs, human umbilical vein endothelial cells; PS, prematurely senescent; RS, replicatively senescent.

Supplementary Table 2. Administration dosages for each candidate compound in high-fat diet-fed obese mice

Compound No.	Injection dose (mg/kg)
HHT (34)	0.545
114	0.426
124	0.176
158	0.359
162	0.219
167	0.832
223	0.996
386	0.751
441	0.502
584	0.633
930	0.985
1242	0.440
1707	0.451

HHT, homoharringtonine

Supplementary Table 3. Median lethal dose (LD50), median effective dose (ED50), and therapeutic index (TI) of HHT and ABT263 in various cell types cultured in 10% FBS containing medium

Compounds		HPAs	HDFs	HUVECs	hRPEs
HHT (34)	LD50[a]	334 nM	208 nM	257 nM	18.2 μM
	ED50[b]	51.6 nM	48.1 nM	279.4 nM	4.23 μM
	TI[c]	6.5 ×	4.3 ×	0.9 ×	4.3 ×
ABT263	LD50	10.2 μM	> 10 μM	1.01 μM	17.8 μM
	ED50	788 nM	665 nM	93.08 nM	5.44 μM
	TI	13 ×	> 15 ×	10.8 ×	3.3 ×

[a]LD50 (median lethal dose): The dose at which 50% of the non-senescent cells exhibit toxic effects.

[b]ED50 (median effective dose): The dose at which 50% of the senescent cells show toxic effects.

[c]TI (therapeutic index) = LD50/ED50.

Replicatively senescent (RS) cells: HPAs, HDFs, and HUVECs.

Doxorubicin-induced prematurely senescent (PS) cells: hRPEs.

HDFs, human dermal fibroblasts; HHT, homoharringtonine; HPAs, human visceral preadipocytes; hRPEs, human retinal pigment epithelial cells; HUVECs, human umbilical vein endothelial cells

Supplementary Table 4. Proteins identified to bind with homoharringtonine based on drug affinity responsive target stability analysis

Putative HHT binding proteins	MW (kDa)
Clathrin heavy chain 1 (CLTC)	180
Alpha-actinin-4, alpha-actinin-1	110
Heat shock protein family A member 5 (HSPA5)	78
Transketolase (TKT)	68
Muscle Pyruvate kinase (PKM2)	58
Thioredoxin reductase 1 (TXNRD1)	55
Retinal dehydrogenase 1 (ALDH1A1)	55
Enolase 1	47
Heat shock 70 kDa protein 8 isoform 2 (HSC54)	70
Glyceraldehyde 3-phosphate dehydrogenase (GAPDH)	37
Uracil DNA glycosylase	35 and 39
Ribosomal protein L7 (RPL7)	30

Supplementary Table 5. Effect of HHT on the complete blood count of *Zmpste24* mice. Male and female 5-week-old *Zmpste24*$^{+/+}$, *Zmpste24*$^{+/-}$, and *Zmpste24*$^{-/-}$ mice were intraperitoneally injected with vehicle (PBS) and HHT for 10 weeks.

Male mice						
	Zmpste24$^{+/+}$		*Zmpste24*$^{+/-}$		*Zmpste24*$^{-/-}$	
	Veh (n=8)	HHT (n=9)	Veh (n=4)	HHT (n=4)	Veh (n=4)	HHT (n=4)
WBC,10^3/μL	8.7 ± 0.98	7.5 ± 0.60	6.4 ± 1.34	6.1 ± 1.18	3.5 ± 1.06	3.9 ± 0.69
RBC,10^6/μL	10.4 ± 0.12	9.5 ± 1.01	10.7 ± 0.20	10.7 ± 0.26	9.9 ± 0.99	10.3 ± 0.23
HGB, g/dL	12.1 ± 0.65	11.9 ± 0.48	12.4 ± 0.82	11.9 ± 0.47	11.8 ± 1.94	12.9 ± 0.85
HCT, %	42.6 ± 0.51	42.3 ± 0.69	43.2 ± 0.94	42.8 ± 1.21	39.3 ± 3.86	40.4 ± 0.86
MCV, fL	40.8 ± 0.16	40.5 ± 0.10	40.5 ± 0.18	40.0 ± 0.22	39.9 ± 0.28	39.1 ± 0.17
MCH, pg	13.3 ± 0.07	13.1 ± 0.05	13.2 ± 0.07	13.0 ± 0.05	13.4 ± 0.19	12.9 ± 0.10
PLT,10^4/μL	5.4 ± 1.98	8.6 ± 2.54	5.9 ± 1.18	7.9 ± 1.31	17.8 ± 6.27	21.4 ± 5.19
NRBC,10^1/μL	2.4 ± 0.60	2.2 ± 0.57	5.5 ± 2.78	0.2 ± 0.20	2.3 ± 0.85	3.4 ± 2.18
NEUT,10^3/μL	1.8 ± 1.10	2.9 ± 0.85	3.6 ± 1.85	5.3 ± 3.16	2.5 ± 1.10	2.7 ± 0.40
LYMPH,10^3/μL	6.4 ± 1.08	4.1 ± 1.01	2.3 ± 0.60	4.3 ± 1.16	0.8 ± 0.12	1.2 ± 0.29
MONO,10^2/μL	1.9 ± 0.26	1.7 ± 0.25	1.1 ± 0.49	0.7 ± 0.17	0.6 ± 0.23	0.6 ± 0.30
EO,10^1/μL	33 ± 4.1	30 ± 3.1	30 ± 10.7	38 ± 19.9	14 ± 7.2	11 ± 3.1
BASO,10^1/μL	1.8 ± 0.25	1.6 ± 0.29	2.3 ± 0.25	2.0 ± 0.58	1.0 ± 0.41	1.3 ± 0.95
**Female mice**						
	Zmpste24$^{+/+}$		*Zmpste24*$^{+/-}$		*Zmpste24*$^{-/-}$	
	Veh (n=3)	HHT (n=5)	Veh (n=4)	HHT (n=5)	Veh (n=4)	HHT (n=6)
WBC,10^3/μL	9.0 ± 0.86	7.6 ± 1.21	8.6 ± 1.27	8.2 ± 1.14	8.9 ± 2.00	6.0 ± 1.59
RBC,10^6/μL	10.4 ± 0.31	10.2 ± 0.25	10.4 ± 0.40	10.4 ± 0.24	10.6 ± 0.27	10.7 ± 0.20
HGB, g/dL	13.8 ± 0.38	13.3 ± 0.35	13.8 ± 0.50	13.6 ± 0.37	14.1 ± 0.40	13.7 ± 0.25
HCT, %	42.1 ± 0.99	40.7 ± 1.00	41.9 ± 1.47	41.4 ± 1.05	42.6 ± 0.85	41.5 ± 1.61
MCV, fL	40.5 ± 0.24	39.6 ± 0.12	40.2 ± 0.24	40.0 ± 0.22	40.0 ± 0.38	38.8 ± 0.30
MCH, pg	13.3 ± 0.13	13.0 ± 0.07	13.3 ± 0.07	13.1 ± 0.15	13.2 ± 0.06	12.8 ± 0.03
PLT,10^4/μL	6.8 ± 2.67	7.3 ± 1.41	4.8 ± 0.71	4.5 ± 0.87	7.4 ± 2.23	10.7 ± 1.71
NRBC, 10^1/μL	7.0 ± 6.00	2.8 ± 1.59	1.3 ± 0.95	4.0 ± 0.45	3.0 ± 1.41	1.8 ± 0.60
NEUT,10^3/μL	0.5 ± 0.10	1.6 ± 0.93	0.5 ± 0.11	0.8 ± 0.15	2.6 ± 0.84	4.9 ± 1.69
LYMPH,10^3/μL	5.4 ± 2.73	5.5 ± 1.50	7.6 ± 1.18	5.4 ± 1.58	5.8 ± 1.74	0.9 ± 0.38
MONO,10^2/μL	1.7 ± 0.32	1.3 ± 0.27	1.7 ± 0.39	1.7 ± 0.31	2.3 ± 0.95	0.7 ± 0.16
EO,10^1/μL	30 ± 4.3	31 ± 2.3	31 ± 2.4	28 ± 3.5	24 ± 3.7	18 ± 5.4
BASO,10^1/μL	2.3 ± 0.33	1.4 ± 0.24	1.8 ± 0.25	2.6 ± 0.68	2.3 ± 0.25	1.3 ± 0.42

Values are presented as means ± SEM. Data were analyzed via one-way analysis of variance (ANOVA) followed by a post-hoc test or via two-tailed Student's *t* test.

BASO, basophils; EO, eosinophils; HCT, hematocrit; HGB, hemoglobin; HHT, homoharringtonine; LYMPH, lymphocytes; MCH, mean corpuscular hemoglobin; MCV, mean corpuscular volume; MONO, monocytes; NEUT, neutrophils; NRBC, nucleated red blood cells; PLT, platelets; RBC, red blood cell; WBC, white blood cell.

Supplementary Table 6. Effect of HHT on the complete blood count of 28-month-old male mice. Male C57BL/6N mice at 16 months were intraperitoneally injected with PBS or HHT once a week for 12 months.

	PBS (n=6)	HHT (n=4)	*t* test
WBC (x10^3/μL)	5.29 ± 0.58	10.49 ± 2.52	0.020
RBC,10^6 μL	7.81 ± 0.40	9.45 ± 0.80	0.037
HGB, g/dL	11.17 ± 0.48	12.43 ± 0.97	0.116
HCT, %	34.00 ± 1.41	39.03 ± 2.90	0.060
MCV, fL	43.63 ± 0.58	41.38 ± 0.89	0.028
MCH, pg	14.32 ± 0.22	13.2 ± 0.39	0.013
MCHC, g/dL	32.83 ± 0.19	31.80 ± 0.32	0.009
PLT,10^4/μL	9.23 ± 2.86	7.45 ± 1.41	0.323
RDW-SD, fL	22.33 ± 0.78	24.48 ± 2.05	0.146
RDW-CV, %	15.13 ± 0.49	18.6 ± 1.79	0.027
MPV, fL	8.75 ± 0.34	8.28 ± 0.24	0.166
P-LCR, %	16.75 ± 3.51	15.00 ± 2.31	0.361
PCT, %	0.13 ± 0.04	0.06 ± 0.01	0.093
NRBC, 10^1/μL	1.17 ± 0.75	5.75 ± 2.17	0.024
NEUT,10^3/μL	2.26 ± 0.70	1.49 ± 0.44	0.216
LYMPH,10^3/μL	2.47 ± 0.69	7.18 ± 2.18	0.020
MONO,10^2/μL	3.22 ± 0.34	14.50 ± 5.44	0.016
EO,10^1/μL	20.33 ± 3.12	34.00 ± 2.61	0.007
BASO,10^1/μL	3.33 ± 1.41	3.00 ± 0.71	0.431

Values are presented as means ± SEM. Data were analyzed via two-tailed Student's *t* test.

BASO, basophils; EO, eosinophils; HCT, hematocrit; HGB, hemoglobin; HHT, homoharringtonine; LYMPH, lymphocytes; MCH, mean corpuscular hemoglobin; MCV, mean corpuscular volume; MONO, monocytes; NEUT, neutrophils; NRBC, nucleated red blood cells; PLT, platelets; RBC, red blood cell; WBC, white blood cell.

Supplementary Table 7. Primary antibodies used in this study

Antibody	Company	Catalog number
Actinin Alpha 4 (ACTN4)	Abcam	Ab59468
Activator protein 1 (AP1)	Novusbio	NBP1-89544
Adipose triglyceride lipase (ATGL)	Cell Signaling Technology	#2138
Aldehyde dehydrogenase 1 family, member A1 (ALDH1A1)	Thermo Fisher Scientific	PA5-34901
β-actin	Gene Tex	GT5512
Caspase-3	Cell Signaling Technology	#9662S
Cleaved caspase-3	Cell Signaling Technology	#9664S
Enolase 1 (ENO1)	Abcam	ab155102
F4/80	Abcam	ab6640
Gamma-H2AX (γH2AX)	Fortis Life Sciences	IHC-00059
Glyceraldehyde 3-phosphate dehydrogenase (GAPDH)	Santa Cruz Biotechnology	sc-25778
Heat shock protein family A member 5 (HSPA5)	LifeSpan Biosciences	LS-C312961
	Cell Signaling Technology	#3177
	Santa Cruz Biotechnology	sc-166490
Heat shock protein family A member 8 (HSPA8)	LifeSpan Biosciences	LS-C312344
Hormone-sensitive lipase (HSL)	Cell Signaling Technology	#4107
His-Taq	Cell Signaling Technology	#12698
iNOS	Abcam	# ab3523
Interleukin-1 beta (IL-1β)	Cell Signaling Technology	#12703, 12242
	Santa Cruz Biotechnology	Sc-1251
Interleukin 6 (IL-6)	Cell Signaling Technology	#12153
Matrix metalloproteinase-2 (MMP2)	Cell Signaling Technology	#87809
Monocyte chemoattractant protein-1 (MCP-1)	Cell Signaling Technology	#39091
p65	Cell Signaling Technology	#3034
Peroxisome proliferator-activated receptor gamma coactivator 1 alpha (PGC1-α)	Abcam	ab54481
phospho-p65	Cell Signaling Technology	#3033
phospho-Rb (pRb)	Cell Signaling Technology	#9308L
Plasminogen activator inhibitor-1 (PAI-1)	Cell Signaling Technology	#11907, 27535
Poly (ADP-ribose) polymerase (PARP)	Santa Cruz Biotechnology	sc-7150
Pyruvate kinase M2 (PKM2)	Cell Signaling Technology	#4053S
p16	Santa Cruz Biotechnology	sc-56330
	Cell Signaling Technology	#29271
p21	Santa Cruz Biotechnology	sc-817
	Cell Signaling Technology	#37543
p53	R&D systems	AF1355
	Abbkine	ABP0110
	Cell Signaling Technology	#2524

Retinoblastoma-associated protein (Rb)	Cell Signaling Technology	#9313T
Ribosomal protein L7 (RPL7)	Abcam	ab72550
Ribosomal Protein S14 (RPS14)	Abcam	ab174661
Thioredoxin reductase 1 (TXNRD1)	Novus Biologicals	NBP2-20619
Transforming growth factor beta 1 (TGF-β1)	Cell Signaling Technology	#3711
Transketolase (TKT)	Thermo Fisher Scientific	PA5-43192
Uncoupling protein 1 (UCP1)	Abcam	ab23841

Supplementary Table 8. CyTOF antibody panel

Cell Type	Antibody	Metal Isotope	Clone	Supplier
CD45+ hematopoietic cells	CD45	89Y	30-F11	Fluidigm
o Lymphoid cells	CD3ε	174Yb	145-2C11	Biolegend
	TCRβ	169Tm	H57-597	Biolegend
	CD4	165Ho	RM4-5	Biolegend
	CD8b	163Dy	H35-17.2	Thermo-Fisher
	CD25	161Dy	3C7	Biolegend
	FoxP3	158Gd	FJK-16s	Thermo-Fisher
	KLRG1	168Er	2F1	BD Biosciences
	CD127	175Lu	A7R34	Biolegend
	CD117	155Gd	2B8	Biolegend
	NK1.1	PK136	PK136	Biolegend
	B220	176Yb	RA3-6B2	Biolegend
	CD19	149Sm	1D3	BD
o Myeloid cells	CD11b	148Nd	M1/70	Biolegend
	F4/80	146Nd	BM8	Fluidigm
	CD206	172Yb	C068C2	Biolegend
	MHCII	144Nd	M5/114.15.2	Biolegend
	CD64	159Tb	X54-5.7.1	Biolegend
	CD11c	142Nd	N418	Biolegend
	CD81	112Cd	B-11	Santa Cruz
	CD36	114Cd	HM36	Biolegend
	CD44	141Pr	IM7	Biolegend
	Siglec-F	143Nd	E50-2440	BD
	Ly6G	167Er	1A8	Biolegend
	Galectin-3*	153Eu	M3/38	Fluidigm
	CD9*	166Er	EM-04	Novus Biologicals
	Ly6c*	115ln	HK1.4	Biolegend
	CD24*	150Nd	M1/69	Biolegend
CD45- stromal cells	CD31	151Eu	390	Biolegend
	Pdgfrα	156Gd	APA5	ThermoFisher
	Pdpln	147Sm	8.1.1	Novus Biologicals
	IL33	171Yb	396118	R&D
	CD34	173Yb	RAM34	ThermoFisher
	Sca1	164Dy	E13-161.7	Biolegend
	DPP4	152Sm	H194-112	Biolegend
	Icam-1	154Sm	YN1/1.7.4	Biolegend
	CD142	160Gd	AF3178	R&D
	Pref1	145Nd	---	R&D

*These markers were also used to interrogate stromal cell populations

Supplementary Table 9. Gene specific primers for RT-qPCR used in the present study

Gene name	Primer sequence	Product size (bp)
Ucp1	Forward: CATCACCACCCTGGCAAAA<br>Reverse: AGCTGATTTGCCTCTGAATGC	80
Prdm16	Forward: ACCTGCCACAGCAAAGAA<br>Reverse: CCATCCAAGCAGAGAAGTAGAC	98
Ppargc1a	Forward: GAATCAAGCCACTACAGACACCG<br>Reverse: CATCCCTCTTGAGCCTTTCGTG	136
Tbx1	Forward: GGCAGGCAGACGAATGTTC<br>Reverse: TTGTCATCTACGGGCACAAAG	102
Tnfrsf9	Forward: GAGGTCAGAAGAGAAAGGGTTG<br>Reverse: GTAGAGGACCCAGGTTTGATTC	97
Cited1	Forward: AACCTTGGAGTGAAGGATCGC<br>Reverse: GTAGGAGAGCCTATTGGAGATGT	128
Gapdh	Forward: CGTTGAATTTGCCGTGAGT<br>Reverse: CAGTGGCAAAGTGGAGATTG	101

Supplementary Table 10. Information about siRNAs used in the present study

siRNA name	Company	siRNA ID	Part Number	Lot#
siPKM2	Life Technologies Corp.	s10574	4390824	ASO2DTL1
siHSPA5	Life Technologies Corp.	s6979	4392420	ASO2DTL4
siHSPA8	Life Technologies Corp.	s6985	4392420	ASO2DTL2
siTXNRD1	Life Technologies Corp.	s755	4390824	ASO2DTL3
siRPL7	Life Technologies Corp.	s352	4390824	ASO2DTL0
Scrambled siRNA (Negative control)	Life Technologies Corp.		4390843	ASO2D2UJ

Supplementary Table 11. Patient information for human adipose tissue samples

Patient number	Sex	Age	Operation name
1	Female	40-49	Pedicled transverse rectus abdominis myocutaneous flap
2	Female	60-69	Pedicled transverse rectus abdominis myocutaneous flap
3	Female	40-49	Pedicled transverse rectus abdominis myocutaneous flap
4	Female	40-49	Pedicled transverse rectus abdominis myocutaneous flap

* No compensation was provided, as the adipose tissue samples were obtained from tissue discarded during pedicled transverse rectus abdominis myocutaneous flap breast reconstruction surgery.

Supplementary Table 12. Small molecule screening data

Category	Parameter	Description
Assay	Type of assay	Cell viability assay (CCK-8) and senescence-associated β-galactosidase (SAβG) staining
	Target	Senescent cells in multiple human cell types (HDFs, HUVECs, hRPEs)
	Primary measurement	Cell survival rate (CCK-8 absorbance), Cell senescence measurement (SAβG positive cell), Cytotoxicity in non-senescent cells
	Key reagents	Cell Counting Kit-8 (CCK-8, Dojindo), SAβG staining solution (X-gal based), Doxorubicin (for premature senescence induction), 10% FBS containing culture media
	Assay protocol	Primary Screen:<br>1. Seed PS HDFs at 1×10^3 cells/well in 96-well plates<br>2. Treat with compounds at 100 nM for 4 days<br>3. Assess viability by CCK-8 and senescence by SAβG staining<br>Secondary Screen:<br>1. Test 110 selected compounds in RS HDFs, RS HUVECs, PS hRPEs<br>2. Same treatment conditions (100 nM, 4 days)<br>3. Test toxicity in non-senescent cells
	Additional comments	Positive controls: ABT263 (senolytic), Rapamycin (senomorphic)
Library	Library size	2,150 compounds
	Library composition	FDA-approved drugs and compounds in phase 1-3 clinical trials
	Source	Korea Chemical Bank (Daejeon, Republic of Korea), https://chembank.org/
	Additional comments	Focus on identifying novel senotherapeutics (senolytics and senomorphics)
Screen	Format	96-well plate format
	Concentration(s) tested	- Primary screen: 100 nM<br>- Secondary validation: 100 nM
	Plate controls	- Positive controls: ABT263, Rapamycin<br>- Negative control: Vehicle (DMSO in PBS)<br>- Non-treated cells
	Reagent/ compound dispensing system	Manual pipetting for compound addition
	Detection instrument and software	- CCK-8: Microplate reader (absorbance measurement)<br>- SAβG: Light microscopy with Leica microsystems
	Assay validation/QC	- Senescence validation: p16, p21 expression by Western blot

		- SAβG positivity: >70% in PS HDFs, >80% in RS HDFs/HUVECs, >60% in PS hRPEs
	Correction factors	Background subtraction for absorbance readings
	Normalization	- Cell viability: % of non-treated control<br>- Protein expression: Normalized to GAPDH
	Additional comments	Three independent experiments performed for each assay
Post-HTS analysis	Hit criteria	Primary screen (2,150 → 110 compounds):<br>- Increased cytotoxicity OR decreased SAβG in PS HDFs<br>Secondary screen (110 → 15 compounds):<br>- Senotherapeutic activity in all 3 cell types (RS HDFs, RS HUVECs, PS hRPEs)<br>- ≥75% viability in non-senescent cells<br>*In vivo* validation (15 → 1 compound):<br>- Reduced body weight gain in HF-fed mice<br>- Improved glucose tolerance<br>- No significant change in food intake
	Hit rate	2,150 → 110 (5.1% of original library) → 15 (0.7% of original library) → 1 (0.05% of original library)
	Additional assay(s)	*In vitro* validation<br>- LDH assay (cytotoxicity confirmation)<br>- Western blot (p53, p21, p16, cleaved caspase-3, PARP)<br>- Flow cytometry (cell cycle analysis)<br>- SASP measurement (IL-1β, IL-6, MCP-1, PAI-1)<br>- Dose-response curves (0-10 μM)<br>*In vivo* validation<br>- HF-induced obesity model (6 wks HF + 8 wks treatment)<br>- IPGTT (glucose tolerance)<br>- ITT (insulin sensitivity)<br>- Tissue analysis (SAβG, histology, Western blot)<br>Mechanism studies<br>- DARTS assay + mass spectrometry<br>- Surface plasmon resonance<br>- siRNA knockdown experiments<br>- ATPase activity assay
	Confirmation of hit purity and structure	- Compound obtained from commercial source (Tocris, Bristol, UK)<br>- Known structure: FDA-approved for CML treatment
	Additional comments	HHT demonstrated:<br>- Senolytic activity in HPAs and HDFs<br>- Senomorphic activity in HUVECs and hRPEs<br>- Cell type-specific effects

	- Improved metabolic function in diet-induced obesity<br>- Extended lifespan in aged and progeroid mice<br>- Target engagement with HSPA5 confirmed by multiple methods
