# Supplementary material for: Homoharringtonine exhibits senotherapeutic activity that mitigates diet- and age-associated obesity and insulin resistance and extends lifespan in mice
Source: Nat Commun. 2026 Mar 31;17:2700. doi: 10.1038/s41467-026-70475-3 (PMC13039285; doi:10.1038/s41467-026-70475-3)
Supplement: Supplementary file 2 — Description of Additional Supplementary Files [file 41467_2026_70475_MOESM2_ESM.pdf]

Description of Additional Supplementary Files

Supplementary Movie 1: HHT improves physical activity of Zmpste24-/- mice. HHT improves physical activity of Zmpste24-/- mice. HHT treatment improves physical activity of Zmpste24-/- mice.

Supplementary Movie 2: HHT improves physical activity of physiological aging mice. HHT improves physical activity of physiological aging mice. HHT treatment improves physical activity of aged mice.
