# Supplementary material for: Homoharringtonine exhibits senotherapeutic activity that mitigates diet- and age-associated obesity and insulin resistance and extends lifespan in mice
Source: Nat Commun. 2026 Mar 31;17:2700. doi: 10.1038/s41467-026-70475-3 (PMC13039285; doi:10.1038/s41467-026-70475-3)
Supplement: Supplementary file 5 — Reporting Summary [file 41467_2026_70475_MOESM5_ESM.pdf]

nature portfolio

Corresponding author(s): Hoon-Ki Sung, Jae-Ryong Kim, So-Young Park

Last updated by author(s): Dec 15, 2025

Reporting Summary

Nature Portfolio wishes to improve the reproducibility of the work that we publish. This form provides structure for consistency and transparency in reporting. For further information on Nature Portfolio policies, see our Editorial Policies and the Editorial Policy Checklist.

Statistics

For all statistical analyses, confirm that the following items are present in the figure legend, table legend, main text, or Methods section.

n/a	Confirmed	
☐	☒	The exact sample size (n) for each experimental group/condition, given as a discrete number and unit of measurement
☐	☒	A statement on whether measurements were taken from distinct samples or whether the same sample was measured repeatedly
☐	☒	The statistical test(s) used AND whether they are one- or two-sided<br>*Only common tests should be described solely by name; describe more complex techniques in the Methods section.*
☒	☐	A description of all covariates tested
☒	☐	A description of any assumptions or corrections, such as tests of normality and adjustment for multiple comparisons
☐	☒	A full description of the statistical parameters including central tendency (e.g. means) or other basic estimates (e.g. regression coefficient) AND variation (e.g. standard deviation) or associated estimates of uncertainty (e.g. confidence intervals)
☐	☒	For null hypothesis testing, the test statistic (e.g. F, t, r) with confidence intervals, effect sizes, degrees of freedom and P value noted<br>*Give P values as exact values whenever suitable.*
☒	☐	For Bayesian analysis, information on the choice of priors and Markov chain Monte Carlo settings
☒	☐	For hierarchical and complex designs, identification of the appropriate level for tests and full reporting of outcomes
☒	☐	Estimates of effect sizes (e.g. Cohen's d, Pearson's r), indicating how they were calculated

Our web collection on statistics for biologists contains articles on many of the points above.

Software and code

Policy information about availability of computer code

Data collection: BIO-RAD Model 680 microplate reader for absorbance measurement; Leica microsystems; Canon EOS 60D digital camera, K1-Fluo Confocal Laser Scanning Microscope, SPECTRAL Ami and Ami X imaging systems, and Fujifilm LAS-3000 image system for image acquisition.LAS-4000 image analyzer (for Western blotting); NanoDrop 2000/2000c (for RNA quantity); Real-Time PCR 7500 System (for qPCR); iNSiGHT VET DXA (for body composition measurement); MLAB (for glucose uptake); CytoFLEX Flow Cytometer (for cell cycle analysis

Data analysis	Graphpad Prism 8 (Graph Pad Software Inc; La Jolla, CA, USA) for data and statistical analysis; ImageJ 1.54p for image analysis. Cell cycle analysis: CytExpert software (Beckman Coulter). Single nuclei data analysis: The 10X Genomics' CellRanger v6.0.0 software was used for sequence alignment and barcode processing with mouse reference genome (mm10-2020-A). Removal of background (non-cellular) barcodes was initially performed using the default CellRanger knee-plot filtering, and potential doublets were removed using 'scDblFinder'. The filtered data was analysed using standard data integration protocol in Seurat v4.0.1, including log normalization, variable feature selection, selection of integration anchors, and Louvain clustering. Our data was mapped on to reference dataset of white adipose tissue in mouse and humans reported by Emont et al (Nature, 2022). Mapping of reference and our (target) datasets was conducted using Seurat's integrated pipeline. Mass spectrometry analysis: Protein spots were excised, digested with trypsin (Promega, Madison, WI), mixed with alpha-cyano-4-hydroxycinnamic acid in 50% acetonitrile /0.1% TFA, and subjected to MALDI-TOF analysis (Microflex LRF 20, Bruker Daltonics) as described Fernandez J et al (Electrophoresis 19: 1036-1045). Spectra were collected from 300 shots per spectrum over m/z range 600-3000 and calibrated by two point internal calibration using Trypsin auto-digestion peaks(m/z 842.5099, 2211.1046). Peak list was generated using Flex Analysis 3.0. Threshold used for peak-picking was as follows: 500 for minimum resolution of monoisotopic mass, 5 for S/N. The search program MASCOT, developed by The Matrixscience (http://www.matrixscience.com/), was used for protein identification by peptide mass fingerprinting. The following parameters were used for the database search: trypsin as the cleaving enzyme, a maximum of one missed cleavage, iodoacetamide (Cys) as a complete modification, oxidation (Met) as a partial modification, monoisotopic masses, and a mass tolerance of ± 0.1 Da. PMF acceptance criteria is probability scoring.

For manuscripts utilizing custom algorithms or software that are central to the research but not yet described in published literature, software must be made available to editors and reviewers. We strongly encourage code deposition in a community repository (e.g. GitHub). See the Nature Portfolio guidelines for submitting code & software for further information.

Data

Policy information about availability of data

All manuscripts must include a data availability statement. This statement should provide the following information, where applicable:

- Accession codes, unique identifiers, or web links for publicly available datasets
- A description of any restrictions on data availability
- For clinical datasets or third party data, please ensure that the statement adheres to our policy

The raw single-nucleus RNA-sequencing (snRNA-seq) data from adipose tissue have been deposited in the NCBI Sequence Read Archive (SRA) under the BioProject accession number PRJNA1260162.

Research involving human participants, their data, or biological material

Policy information about studies with human participants or human data. See also policy information about sex, gender (identity/presentation), and sexual orientation and race, ethnicity and racism.

Reporting on sex and gender	All fat tissue samples are obtained from female sex and gender.
Reporting on race, ethnicity, or other socially relevant groupings	All fat tissue samples are obtained from Asian (Korean).
Population characteristics	All human subcutaneous adipose tissues were obtained from patients who underwent mammoplasty after mastectomy due to breast cancer.
Recruitment	All patients declared their informed consent in written form.
Ethics oversight	This study has been approved by the IRB at the Yeungnam University Medical Center (YUMC 2021-03-052).

Note that full information on the approval of the study protocol must also be provided in the manuscript.

Field-specific reporting

Please select the one below that is the best fit for your research. If you are not sure, read the appropriate sections before making your selection.

☒ Life sciences ☐ Behavioural & social sciences ☐ Ecological, evolutionary & environmental sciences

For a reference copy of the document with all sections, see nature.com/documents/nr-reporting-summary-flat.pdf

Life sciences study design

All studies must disclose on these points even when the disclosure is negative.

Sample size	No sample size calculation was performed. Sample size was determined by similar studies in the field, as well as our previous reports.
Data exclusions	Mice were excluded from the study when exhibiting apparent sickness, weight loss, and in-cage aggression.
Replication	All replication attempts were successful.

Randomization	Age and weight-mached mice were randomly allocated into PBS or HHT groups. For in vitro experiments, cell culture wells were also randomly allocated into PBS or HHT treatment groups.
Blinding	All samples from mice were labeled with numerical code.

Reporting for specific materials, systems and methods

We require information from authors about some types of materials, experimental systems and methods used in many studies. Here, indicate whether each material, system or method listed is relevant to your study. If you are not sure if a list item applies to your research, read the appropriate section before selecting a response.

Materials & experimental systems

n/a	Involved in the study
☐	☒ Antibodies
☐	☒ Eukaryotic cell lines
☒	☐ Palaeontology and archaeology
☐	☒ Animals and other organisms
☒	☐ Clinical data
☒	☐ Dual use research of concern
☒	☐ Plants

Methods

n/a	Involved in the study
☒	☐ ChIP-seq
☐	☒ Flow cytometry
☒	☐ MRI-based neuroimaging

Antibodies

Antibodies used

1. Actinin Alpha 4(ACTN4), Abcam, ab59468
2. Activator protein 1 (AP1), Novusbio, NBP1-89544
3. Adipose triglyceride lipase (ATGL), Cell Signaling Technology, #2138
4. Aldehyde dehydrogenase 1 family, member A1(ALDH1A1), Thermo Fisher Scientific, PA5-34901
5. β-actin, Gene Tex, GT5512
6. Caspase-3, Cell Signaling Technology, #9662S
7. Cleaved caspase-3, Cell Signaling Technology, #9664S
8. Enolase 1(ENO1), Abcam, ab155102
9. F4/80, Abcam, ab6640
10. Gamma-H2AX (H2AX), Fortis Life Sciences, IHC-00059
11. Glyceraldehyde 3-phosphate dehydrogenase (GAPDH), Santa Cruz Biotechnology, sc-25778
12. Heat Shock 70kDa Protein 5(HSPA5), LifeSpan Biosciences, LS-C312961
13. Heat Shock 70kDa Protein 5(HSPA5), Cell Signaling Technology, #3177
14. Heat Shock 70kDa Protein 5(HSPA5), Santa Cruz Biotechnology, sc-166490
15. Heat shock 70 kDa protein 8(HSPA8), LifeSpan Biosciences, LS-C312344
16. Hormone-sensitive lipase (HSL), Cell Signaling Technology, #4107
17. His-Taq, Cell Signaling Technology, #12698
18. iNOS, Abcam, # ab3523
19. Interleukin-1 beta (IL-1β), Cell Signaling Technology, #12703
20. Interleukin-1 beta (IL-1β), Cell Signaling Technology, #12242
21. Interleukin-1 beta (IL-1β), Santa Cruz Biotechnology, sc-1251
22. Interleukin 6 (IL-6), Cell Signaling Technology , #12153
23. Matrix metalloproteinase-2 (MMP2), Cell Signaling Technology, #87809
24. Monocyte chemoattractant protein-1, Cell Signaling Technology, #39091
25. p65, Cell Signaling Technology, #3034
26. Peroxisome proliferator-activated receptor gamma coactivator 1 alpha (PGC1-α), Abcam , ab54481
27. phospho-p65, Cell Signaling Technology, #3033
28. phospho-Rb (pRb), Cell Signaling Technology, #9308L
29. Plasminogen activator inhibitor-1(PAI-1), Cell Signaling Technology, #11907
30. Plasminogen activator inhibitor-1(PAI-1), Cell Signaling Technology, #27535
31. Poly (ADP-ribose) polymerase(PARP), Santa Cruz Biotechnology, sc-7150
32. Pyruvate kinase M2(PKM2), Cell Signaling Technology, #4053S
33. p16, Santa Cruz Biotechnology, sc-56330
34. p16, Cell Signaling Technology, #29271
35. p21, Santa Cruz Biotechnology, sc-817
36. p21, Cell Signaling Technology, #37543
37. p53, R&D systems, AF1355
38. p53, Abbkine, ABP0110
39. p53, Cell Signaling Technology, #2524
40. Retinoblastoma-associated protein (Rb), Cell Signaling Technology, #9313T
41. Ribosomal protein L7(RPL7), Abcam, ab72550
42. Ribosomal Protein S14 (RPS14), Abcam, ab174661
43. Thioredoxin reductase 1(TXNRD1), Novus Biologicals, NBP2-20619
44. Transforming growth factor beta 1 (TGF-β1), Cell Signaling Technology, #3711

45. Transketolase(TKT), Thermo Fisher Scientific, PA5-43192
46. Uncoupling protein 1 (UCP1), Abcam, ab23841

Validation

All antibodies utilized in this research were acquired from commercial suppliers, and their validation statements can be found on the manufactur's website. The antibodies were validated by immunostaining or western blot analysis.
1. Actinin Alpha 4(ACTN4), Abcam, ab59468
https://www.abcam.com/en-us/products/primary-antibodies/alpha-actinin-4-antibody-cytoskeleton-marker-ab59468
2. Activator protein 1 (AP1), Novusbio, NBP1-89544
https://www.novusbio.com/products/junb-ap-1-antibody_nbp1-89544?srsltid=AfmBOopyQ32u0DW9lx9wWh0OWAnCmROwUD4JuIPRUBNKCAvSNzGlYzTu
3. Adipose triglyceride lipase (ATGL), Cell Signaling Technology, #2138
https://www.cellsignal.com/products/primary-antibodies/atgl-antibody/2138?srsltid=AfmBOoqSh3-ACWB6o5wscyd8Rj_c1sOMDb5P0FOcDfuNBCwWU4SPsVJR
4. Aldehyde dehydrogenase 1 family, member A1(ALDH1A1), Thermo Fisher Scientific, PA5-34901
https://www.thermofisher.com/antibody/product/ALDH1A1-Antibody-Polyclonal/PA5-34901
5. β-actin, Gene Tex, GT5512
https://www.genetex.com/Product/Detail/beta-Actin-antibody-GT5512/GTX629630?srsltid=AfmBOoq0v_Q-4jW--UojnjlK7kNYjYJBwB-_jlLoDPDxtZcBzaKxiVAk
6. Caspase-3, Cell Signaling Technology, #9662S
https://www.cellsignal.com/products/primary-antibodies/caspase-3-antibody/9662?srsltid=AfmBOor4CkktXB5ARRmx6zhCj5UpfHS9QAU1W1kYjO1gzIldYLINzErX
7. Cleaved caspase-3, Cell Signaling Technology, #9664S
https://www.cellsignal.com/products/primary-antibodies/cleaved-caspase-3-asp175-5a1e-rabbit-monoclonal-antibody/9664?srsltid=AfmBOoqf_0INiRo-dFIbHDEtcDE2IXwb1zdUATG7t0BnuXAdKLTyUuaR
8. Enolase 1(ENO1), Abcam, ab155102
https://www.abcam.com/en-us/products/primary-antibodies/eno1-eno2-eno3-antibody-epr10863b-ab155102
9. F4/80, Abcam, ab6640
https://www.abcam.com/en-us/products/primary-antibodies/f4-80-antibody-cia3-1-macrophage-marker-ab6640
10. Gamma-H2AX (H2AX), Fortis Life Sciences, IHC-00059
https://www.fortislife.com/products/primary-antibodies/rabbit-anti-gamma-h2ax-ihc-antibody/BETHYL-IHC-00059
11. Glyceraldehyde 3-phosphate dehydrogenase (GAPDH), Santa Cruz Biotechnology, sc-25778
https://www.scbt.com/p/gapdh-antibody-fl-335?srsltid=AfmBOopZn-wgEBEm0V8o_nxnngdMu_lIbOLdTeeVTlqvTClQ9TUjo-PA
12. Heat Shock 70kDa Protein 5(HSPA5), LifeSpan Biosciences, LS-C312961
https://www.lsbio.com/antibodies/hspa5-antibody-grp78-antibody-bip-antibody-aa603-617-icc-ihc-wb-western-ls-c312961/322933
13. Heat Shock 70kDa Protein 5(HSPA5), Cell Signaling Technology, #3177
https://www.cellsignal.com/products/primary-antibodies/bip-c50b12-rabbit-monoclonal-antibody/3177?srsltid=AfmBOoqAhVbWlYoQF3g3LJytL4UknlaApXiZWiZDauz3wpv-_bZj9Ptz
14. Heat Shock 70kDa Protein 5(HSPA5), Santa Cruz Biotechnology, sc-166490
https://www.scbt.com/p/grp-78-antibody-e-4?srsltid=AfmBOorqNYe7RX8aFQsq60xn1zYo28EnHybLSrmMgRK-jSyGldyuJ6N6
15. Heat shock 70 kDa protein 8(HSPA8), LifeSpan Biosciences, LS-C312344
https://www.lsbio.com/search?q=Heat+shock+70+kDa+protein+8%28HSPA8%29%2C++LS-C312344
16. Hormone-sensitive lipase (HSL), Cell Signaling Technology, #4107
https://www.cellsignal.com/products/primary-antibodies/hsl-antibody/4107?srsltid=AfmBOorxJsGNbqiKL_8Uklj2kce-UBdfgvku6CQwFcm0IBQqjPCBd2iu
17. His-Taq, Cell Signaling Technology, #12698
https://www.cellsignal.com/products/primary-antibodies/his-tag-d3i1o-rabbit-monoclonal-antibody/12698?srsltid=AfmBOopNdH3TV3HMGq6omsqnKOoWGK_QvUUBAU-zulhA8feEFwTYconO
18. iNOS, Abcam, # ab3523
https://www.abcam.com/en-us/products/primary-antibodies/inos-antibody-ab3523
19. Interleukin-1 beta (IL-1β), Cell Signaling Technology, #12703
https://www.cellsignal.com/products/primary-antibodies/il-1-beta-d3u3e-rabbit-monoclonal-antibody/12703?srsltid=AfmBOoo2-T1si00jSr1Os69b3hibNJCuioCa0SDx7b_LLBZQb0uI40e-
20. Interleukin-1 beta (IL-1β), Cell Signaling Technology, #12242
https://www.cellsignal.com/products/primary-antibodies/il-1-beta-3a6-mouse-monoclonal-antibody/12242?srsltid=AfmBOorjfoTRXW8qVS-YthtqEAU5KrNAtB6ecxueDPCxLok5TpYysFo-
21. Interleukin-1 beta (IL-1β), Santa Cruz Biotechnology, sc-1251
https://www.scbt.com/p/il-1beta-antibody-m-20?srsltid=AfmBOopH0thryCiZwo7M8FHFKLfP70HBzEzZQKe1LBA5px8S7Usv1HhQ
22. Interleukin 6 (IL-6), Cell Signaling Technology , #12153
https://www.cellsignal.com/products/primary-antibodies/il-6-d3k2n-rabbit-monoclonal-antibody/12153?srsltid=AfmBOoooJEJTuG0pLJ4bFBxqRw7h0VipmiGKXi83-4orO1T5_AmhL4ZL
23. Matrix metalloproteinase-2 (MMP2), Cell Signaling Technology, #87809
https://www.cellsignal.com/products/primary-antibodies/mmp-2-d2o4t-rabbit-monoclonal-antibody/87809?srsltid=AfmBOoqmhEZjQPCHEM2a-9a7ULbiSpbTat_TDOEWWKz00CwlUXFINwmY
24. Monocyte chemoattractant protein-1, Cell Signaling Technology, #39091
https://www.cellsignal.com/products/primary-antibodies/mcp-1-antibody-carboxy-terminal-antigen/39091?srsltid=AfmBOoqhcmtXXgcFqqXPwGcnVrZLRhXTjGudMVdGBnhFWb-HQUlGspFa
25. p65, Cell Signaling Technology, #3034
https://www.cellsignal.com/products/primary-antibodies/nf-kb-p65-antibody/3034?srsltid=AfmBOorO_m3GYqv-KQdGmTp0OAROfK_STcTieqc_3PwcHrCsx4hO-tcw
26. Peroxisome proliferator-activated receptor gamma coactivator 1 alpha (PGC1-α), Abcam , ab54481
https://www.abcam.com/en-us/products/primary-antibodies/pgc1-alpha-beta-antibody-bsa-and-azide-free-ab54481
27. phospho-p65, Cell Signaling Technology, #3033
https://www.cellsignal.com/products/primary-antibodies/phospho-nf-kappab-p65-ser536-93h1-rabbit-monoclonal-antibody/3033?srsltid=AfmBOooH-6i7j6zujOBJ555A8SXyHPE6GhrduE6-ZTBv089oLAxWKTN6
28. phospho-Rb (pRb), Cell Signaling Technology, #9308L
https://www.cellsignal.com/products/primary-antibodies/phospho-rb-ser807-811-antibody/9308?

srsltid=AfmBOopLYBl1ytyHzepoNn3IbksKttXNLpFQYb-V0QNDKV42c3-Pkuoz
29. Plasminogen activator inhibitor-1(PAI-1), Cell Signaling Technology, #11907
https://www.cellsignal.com/products/primary-antibodies/pai-1-d9c4-rabbit-monoclonal-antibody/11907?
srsltid=AfmBOorhGh6R_qph7KK4Cf3tqmNMjOOvtjBYfrkToAQ14ROfwPWh9kqQ
30. Plasminogen activator inhibitor-1(PAI-1), Cell Signaling Technology, #27535
https://www.cellsignal.com/products/primary-antibodies/pai-1-antibody/27535?
srsltid=AfmBOoo4d2ZYiMMsadqyGb8ST15xBNHPD7dJTYhQ-8s6wldcrIn0muUE
31. Poly (ADP-ribose) polymerase(PARP), Santa Cruz Biotechnology, sc-7150
https://www.scbt.com/p/parp-1-antibody-h-250?srsltid=AfmBOopaojVlRsjkUGj_UMkLMpCw6tvwdzHtEs1K_1eGdIipyLboDzFN
32. Pyruvate kinase M2(PKM2), Cell Signaling Technology, #4053S
https://www.cellsignal.com/products/primary-antibodies/pkm2-d78a4-rabbit-monoclonal-antibody/4053?
srsltid=AfmBOorXOa6QqHFLo2E6i0q_dsAf6wwaPY0W35ZjSb-42mXn3oY5OL9u
33. p16, Santa Cruz Biotechnology, sc-56330
https://www.scbt.com/p/p16-antibody-jc8?srsltid=AfmBOoo7JIHBKOZYZvLhnoFafe9hJrnu-8M3SiNGMtjMjW4tfXv3hM7N
34. p16, Cell Signaling Technology, #29271
https://www.cellsignal.com/products/primary-antibodies/p16-ink4a-e5f3y-rabbit-monoclonal-antibody/29271?
srsltid=AfmBOooxB0JimNY0xxoqa-rzByJmp7-erKcZKvmpxFmq5C2bqFlAMaLN
35. p21, Santa Cruz Biotechnology, sc-817
https://www.scbt.com/p/p21-antibody-187?srsltid=AfmBOopGlMKoBlw5JHqzWDyD-oj1FTiQ5HB9jDpdUyzsUartl4-LzleD
36. p21, Cell Signaling Technology, #37543
https://www.cellsignal.com/products/primary-antibodies/p21-waf1-cip1-e2r7a-rabbit-monoclonal-antibody/37543?
srsltid=AfmBOoplX9IABOTAkGV3RGie5ojwrAIpfAKquhCLkPvmHI0pcCqNAP8_
37. p53, R&D systems, AF1355
https://www.rndsystems.com/products/human-mouse-rat-p53-antibody_af1355
38. p53, Abbkine, ABP0110
https://www.abbkine.com/abbkine-p53-antibody-helps-basic-cancer-research/
39. p53, Cell Signaling Technology, #2524
https://www.cellsignal.com/products/primary-antibodies/p53-1c12-mouse-monoclonal-antibody/2524?
srsltid=AfmBOordlleKci71Q9riwKPmiRfOdIzq3yYW5EuvOIzVTUnWJfFHDqB8
40. Retinoblastoma-associated protein (Rb), Cell Signaling Technology, #9313T
https://www.cellsignal.com/products/primary-antibodies/rb-d20-rabbit-monoclonal-antibody/9313?
srsltid=AfmBOoqu6k32P5wmrKF0gtlRlcpQzcM-v21EeSN_1Uz32_3B09IH1Z3R
41. Ribosomal protein L7(RPL7), Abcam, ab72550
https://www.abcam.com/en-us/products/primary-antibodies/rpl7-antibody-ab72550
42. Ribosomal Protein S14 (RPS14), Abcam, ab174661
https://www.abcam.com/en-us/products/primary-antibodies/rps14-antibody-ab174661
43. Thioredoxin reductase 1(TXNRD1), Novus Biologicals, NBP2-20619
https://www.novusbio.com/products/thioredoxin-reductase-1-trxr1-antibody_nbp2-20619?
srsltid=AfmBOoqwVqqdytsO32lbHqYxUxEW7Koc20DhbEy0BdzMZLdibPViSdr5
44. Transforming growth factor beta 1 (TGF-β1), Cell Signaling Technology, #3711
https://www.cellsignal.com/products/primary-antibodies/tgf-beta-antibody/3711?
srsltid=AfmBOooC9H9sVB2_0TVZDGrBa1R0TVF7eK28CS-ZhWG-FSOgc9awVQU5
45. Transketolase(TKT), Thermo Fisher Scientific, PA5-43192
https://www.thermofisher.com/antibody/product/Transketolase-Antibody-Polyclonal/PA5-43192
46. Uncoupling protein 1 (UCP1), Abcam, ab23841
https://www.abcam.com/en-us/products/primary-antibodies/ucp1-antibody-ab23841

Eukaryotic cell lines

Policy information about cell lines and Sex and Gender in Research

Cell line source(s)	Human visceral preadipocytes (PT-5005) and human umbilical vein endothelial cells (C2517A) were purchased from Lonza (Basel, Switzerland). Human dermal fibroblasts (PCS-201-012), human retinal pigment epithelial cells (CRL-2302) and 3T3-L1 mouse preadipocytes (CL-173) were purchased from the American Type Culture Collection (ATCC) (VA, USA).
Authentication	HPAs and HUVECs were authenticated by Lonza, and HDFs, hRPEs, and 3T3-L1 cells by ATCC. Preadipocytes identity was confirmed by adipogenic differentiation, while HDFs, HUVECs, and hRPEs authentication was verified by Western blot analysis using cell type–specific protein markers, most recently in October 2024.
Mycoplasma contamination	Cells were tested for mycoplasma contamination by the vendor (all mycoplasma negative).
Commonly misidentified lines (See ICLAC register)	n/a

Animals and other research organisms

Policy information about studies involving animals; ARRIVE guidelines recommended for reporting animal research, and Sex and Gender in Research

Laboratory animals	C57BL/6J and C57BL/6N male, 8 weeks-16 months of age; p16-luc, 8 weeks of age; Zmpste24-/- progeroid mice, 5 weeks of age
Wild animals	Wild animals were not used for this study

Reporting on sex	Mice used in this study were males except Zmpste24-/- progeroid mice (both male and female data are collected).
Field-collected samples	This study does not involve field-collected samples.
Ethics oversight	All animal use protocols were approved by Institutional Animal Care and Use Committee of Yeungnam University College of Medicine. (YUMC-AEC2019-031, YUMC-AEC2020-025, and YUMC-AEC2022-039) and by the Animal Care Committee of The Centre for Phenogenomics (TCP, AUP #27-0271H).

Note that full information on the approval of the study protocol must also be provided in the manuscript.

Flow Cytometry

Plots

Confirm that:

☐ The axis labels state the marker and fluorochrome used (e.g. CD4-FITC).

☒ The axis scales are clearly visible. Include numbers along axes only for bottom left plot of group (a 'group' is an analysis of identical markers).

☐ All plots are contour plots with outliers or pseudocolor plots.

☒ A numerical value for number of cells or percentage (with statistics) is provided.

Methodology

Sample preparation	Cells were harvested and fixed in 70% cold ethanol at 4°C for 1 h. Cells were washed twice with PBS and resuspended in a propidium iodide solution. RNase A was added to a final concentration of 0.5 µg/mL, and the samples were incubated at 4°C for 4 h.
Instrument	Flow cytometric analysis was performed using a CytoFLEX Flow Cytometer (Beckman Coulter Life Sciences, Brea, CA, USA).
Software	Data acquisition and cell cycle analysis were conducted using CytExpert software (Beckman Coulter).
Cell population abundance	Cell cycle distribution was expressed as the percentage of cells in the G0/G1, S, and G2/M phases relative to the total analyzed cell population. At least 10,000 single-cell events were collected per sample to ensure statistical reliability.
Gating strategy	Initial gating was performed on a forward scatter (FSC) versus side scatter (SSC) plot to exclude debris and select the main cell population. Doublets and cell aggregates were excluded by gating on FSC-H versus FSC-A. DNA content histograms of PI fluorescence were then used to determine the proportions of cells in G0/G1, S, and G2/M phases.

☐ Tick this box to confirm that a figure exemplifying the gating strategy is provided in the Supplementary Information.
